# Associations of Age at Diagnosis and Duration of Diabetes With Morbidity and Mortality Among Older Adults

**DOI:** 10.1001/jamanetworkopen.2022.32766

**Published:** 2022-09-30

**Authors:** Christine T. Cigolle, Caroline S. Blaum, Chen Lyu, Jinkyung Ha, Mohammed Kabeto, Judy Zhong

**Affiliations:** 1 Department of Family Medicine, University of Michigan, Ann Arbor; 2 Department of Internal Medicine, University of Michigan, Ann Arbor; 3 Veterans Affairs Ann Arbor Healthcare System Geriatric Research, Education and Clinical Center, Ann Arbor, Michigan; 4 Department of Medicine, New York University Langone Health, New York, New York; 5 Division of Biostatistics, Department of Population Health, NYU Grossman School of Medicine, New York, New York

## Abstract

**Importance:**

Older adults vary widely in age at diagnosis and duration of type 2 diabetes, but treatment often ignores this heterogeneity.

**Objectives:**

To investigate the associations of diabetes vs no diabetes, age at diagnosis, and diabetes duration with negative health outcomes in people 50 years and older.

**Design, Setting, and Participants:**

This cohort study included participants in the 1995 through 2018 waves of the Health and Retirement Study (HRS), a population-based, biennial longitudinal health interview survey of older adults in the US. The study sample included adults 50 years or older (n = 36 060) without diabetes at entry. Data were analyzed from June 1, 2021, to July 31, 2022.

**Exposures:**

The presence of diabetes, specifically the age at diabetes diagnosis, was the main exposure of the study. Age at diagnosis was defined as the age when the respondent first reported diabetes. Adults who developed diabetes were classified into 3 age-at-diagnosis groups: 50 to 59 years, 60 to 69 years, and 70 years and older.

**Main Outcomes and Measures:**

For each diabetes age-at-diagnosis group, a propensity score–matched control group of respondents who never developed diabetes was constructed. The association of diabetes with the incidence of key outcomes—including heart disease, stroke, disability, cognitive impairment, and all-cause mortality—was estimated and the association of diabetes vs no diabetes among the age-at-diagnosis case and matched control groups was compared.

**Results:**

A total of 7739 HRS respondents developed diabetes and were included in the analysis (4267 women [55.1%]; mean [SD] age at diagnosis, 67.4 [9.9] years). The age-at-diagnosis groups included 1866 respondents at 50 to 59 years, 2834 at 60 to 69 years, and 3039 at 70 years or older; 28 321 HRS respondents never developed diabetes. Age at diagnosis of 50 to 59 years was significantly associated with incident heart disease (hazard ratio [HR], 1.66 [95% CI, 1.40-1.96]), stroke (HR, 1.64 [95% CI, 1.30-2.07]), disability (HR, 2.08 [95% CI, 1.59-2.72]), cognitive impairment (HR, 1.30 [95% CI, 1.05-1.61]), and mortality (HR, 1.49 [95% CI, 1.29-1.71]) compared with matched controls, even when accounting for diabetes duration. These associations significantly decreased with advancing age at diagnosis. Respondents with diabetes diagnosed at 70 years or older only showed a significant association with the outcome of elevated mortality (HR, 1.08 [95% CI, 1.01-1.17]).

**Conclusions and Relevance:**

The findings of this cohort study suggest that age at diabetes diagnosis was differentially associated with outcomes and that younger age groups were at elevated risk of heart disease, stroke, disability, cognitive impairment, and all-cause mortality. These findings reinforce the clinical heterogeneity of diabetes and highlight the importance of improving diabetes management in adults with earlier diagnosis.

## Key Points

**Question:**

Is age at diabetes diagnosis associated with the risk of distal outcomes among adults 50 years and older?

**Findings:**

This cohort study of 36 060 adults 50 years and older used data from a national longitudinal health survey and found that diabetes diagnosed at 50 to 59 years of age was significantly associated with elevated risks of incident heart disease, stroke, disability, cognitive impairment, and mortality. However, the associations between diabetes and all outcomes decreased as age at diabetes diagnosis increased, even when accounting for diabetes duration.

**Meaning:**

Differences in the association of diabetes with negative health outcomes support differential diabetes management across age-at-diagnosis groups.

## Teaser

This cohort study assesses the association of age at diabetes diagnosis with heart disease, stroke, disability, cognitive impairment, and all-cause mortality among US adults aged 50 years and older.

## Introduction

Older adults with diabetes are a distinctive age group within the broader population of individuals with diabetes. Demographically, older adults form an expanding segment of the US population; epidemiologically, diabetes has its greatest prevalence among older adults.^1^ Clinically, older adults with diabetes are heterogeneous, varying widely in age, age at diagnosis of diabetes, and duration of diabetes.^2^ However, older adults with diabetes are often treated similarly to middle-aged adults with diabetes, and there is not yet consensus regarding the best management for this population.^3,4,5,6,7,8^

Improving disease management and determining high-quality care for older adults with diabetes requires a clearer understanding of the associations of age, age at diagnosis, and diabetes duration with diabetes-related outcomes. A person with newly diagnosed diabetes at 50 years of age differs in many ways at baseline and over time (eg, 10 years after diagnosis) from a person with newly diagnosed diabetes at 70 years of age. We hypothesized that age at diabetes diagnosis would have a differential association with the risk of incident distal outcomes while accounting for diabetes duration. Evidence from randomized clinical trials^8^ and observational studies^9,10^ suggests that there is heterogeneity in the risk and development of distal outcomes in individuals with diabetes and that diabetes may have a differential effect among older adults compared with younger adults.^10^

There are 3 challenges when testing our hypothesis. First, comorbid conditions present at diabetes diagnosis must be distinguished from comorbid conditions that develop subsequent to diabetes (ie, prevalent comorbidities vs incident diabetes outcomes). As the duration of diabetes increases, heterogeneity of diabetes outcomes will be associated with differences in both baseline and postbaseline characteristics. Such differences could include differences in prevalent comorbidities at baseline and/or different exposures associated with diabetes at baseline and going forward, including variable levels of hyperglycemia and mechanisms of diabetes physiology (eg, beta cell loss or decreased function, insulin resistance) and different patterns of obesity, behaviors, and treatment exposures.^11^ Second, it is important to distinguish aging itself from age at diabetes diagnosis, because both increase the incidences of distal outcomes.^12^ Third, when drawing on evidence from randomized clinical trials and observational studies, it is important to make a distinction between incident diabetes and prevalent diabetes. Much of the current evidence from clinical trials involving older adults addresses prevalent diabetes^13,14,15,16,17^; however, less is known about the effect of age at diagnosis of diabetes (ie, age at diabetes incidence) on distal outcomes in this population.

In this cohort study, we used longitudinal, population-based survey data during a 23-year interval to investigate 5 distal diabetes-related outcomes important in the older adult population: heart disease, stroke, disability, cognitive impairment, and all-cause mortality. We examined 3 age-at-diagnosis groups: 50 to 59 years, 60 to 69 years, and 70 years or older. We compared these participants with newly diagnosed diabetes with their corresponding age group–matched and propensity score–matched controls without diabetes across multiple years.

## Methods

### Data and Study Design

In this cohort study, we performed a secondary analysis of data from the 1995 to 2018 waves of the Health and Retirement Study (HRS). The HRS is a nationally representative, biennial longitudinal health interview survey of middle-aged and older adults in the US.^18^ All respondents provide informed consent on their entry into the HRS. The HRS is sponsored by the National Institute on Aging and is performed by the Institute for Social Research at the University of Michigan, Ann Arbor; it has been approved by the University of Michigan Health Sciences Institutional Review Board. The data used in this analysis are publicly available and contain no unique identifiers, thus ensuring respondent anonymity. This study followed the Strengthening the Reporting of Observational Studies in Epidemiology (STROBE) reporting guideline.

Our study included respondents from 6 HRS study cohorts (encompassing those born before 1924 to those born in 1959).^18^ Respondents included adults living in the community and those in long-stay nursing facilities. When the respondent was unable to be interviewed for a survey wave, a proxy respondent—usually a spouse— answered questions according to HRS protocol.^18^

**Figure 1.**
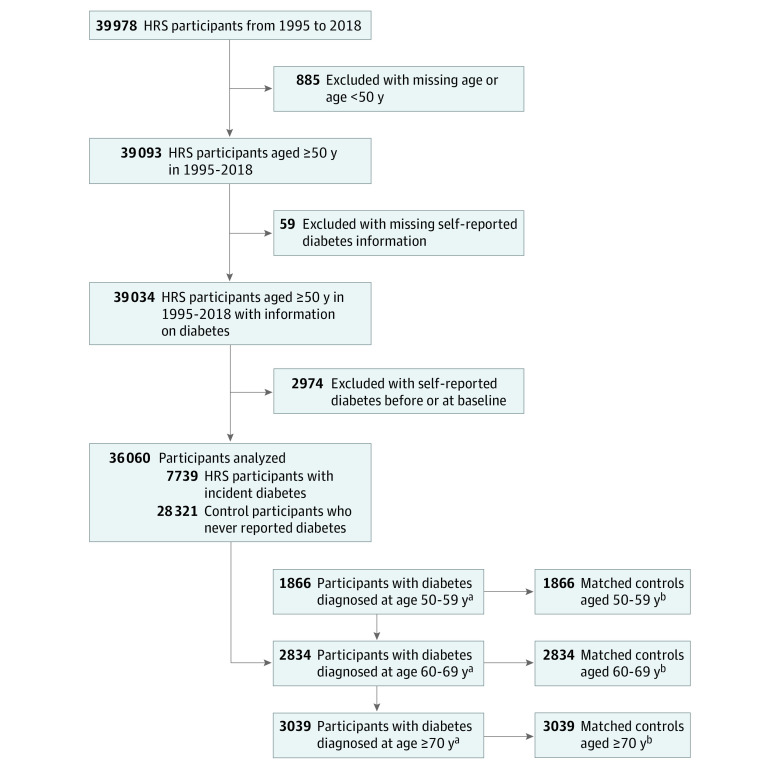
Flowchart of the Study Design HRS indicates Health and Retirement Study. ^a^For each outcome, we further excluded individuals with missing outcomes or with an outcome before diabetes diagnosis. The sample size for each outcome is listed in eTable 2 in the Supplement. ^b^Matched 1:1 by propensity score constructed by HRS birth cohort, sex, race and ethnicity, educational attainment, calendar year of diabetes diagnosis, age, marital status, wealth, body mass index, activities of daily living and instrumental activities of daily living score, cognitive status, and comorbid conditions during the calendar year of diabetes diagnosis. Matched controls were only identified for individuals with diabetes with complete covariates during the calendar year of diabetes diagnosis. The sample sizes of the individuals with diabetes and complete covariates and the matched controls are listed in eTable 2 in the Supplement for each outcome.

We included respondents who self-reported incident diabetes with a diagnosis at 50 years or older and respondents who never reported diabetes during their HRS interviews (controls) (Figure 1). Among the 39 978 HRS respondents from 1995 to 2018, we excluded 885 individuals with missing age data or who were younger than 50 years, 59 individuals with missing self-reported diabetes information, and 2974 individuals who reported diabetes at their baseline HRS interviews (ie, who had prevalent diabetes). The analysis cohort thus consisted of 36 060 respondents: 7739 with self-reported incident diabetes at some time in their subsequent HRS interviews and 28 321 who never reported diabetes at any of their following HRS interviews. Of the 7739 respondents with incident diabetes, 1866 had a diagnosis at 50 to 59 years of age; 2834, at 60 to 69 years of age; and 3039, at 70 years or older. For each outcome, we further excluded the individuals who had that outcome before their diagnosis of diabetes (ie, prevalent condition) or who had missing data for that outcome. The sample sizes for each of the outcomes are included in eTable 2 in the Supplement.

Controls were matched 1:1 by propensity scores constructed using age, sex, race and ethnicity, marital status, educational level, net worth, HRS birth cohort, calendar year of diabetes diagnosis, body mass index (BMI; calculated as weight in kilograms divided by height in meters squared), activities of daily living and instrumental activities of daily living (ADL-IADL) score, cognitive status, and comorbid conditions present in the calendar year of diabetes diagnosis. Matched controls were only identified for individuals with diabetes and complete covariate information in the calendar year of diabetes diagnosis. The sample sizes of the respondents with diabetes and complete covariate data and their matched controls are listed in eTable 2 in the Supplement for each outcome.

### Variables and Their Measurements

#### Diabetes Exposure

The presence of diabetes, specifically the age at which diabetes was diagnosed, was the main exposure. Each HRS biennial core survey wave provides self-reported health conditions, including diabetes. Respondents self-reported whether or not a physician diagnosed them with each disease. Self-report of diabetes was adjudicated using a methodology (eMethods in the Supplement) to address any inconsistency in the self-report of chronic diseases across HRS survey waves and thereby to improve disease classification across waves.^19^ The year of and age at diabetes diagnosis (incidence) were defined as the year and age when the respondent first reported diabetes, preceded by “no diabetes” in previous wave(s). We classified the respondents with diabetes into 3 age-at-diagnosis groups: 50 to 59 years, 60 to 69 years, and 70 years and older. Respondents who never reported having diabetes during the analyzed HRS follow-up duration formed the control group.

#### Outcomes

We investigated 5 incident diabetes-related outcomes: heart disease, stroke, disability, cognitive impairment, and mortality. Heart disease and stroke were measured using the self-report of heart disease and stroke in the HRS core survey and were adjudicated using the same methodology as for diabetes.^19^ The year and age at incident heart disease or stroke were defined as the year and age when a respondent first reported these outcomes. Disability was measured using self-reported ADL dependencies (walking across a room, dressing, bathing, eating, getting in and out of bed, using the toilet) and IADL dependencies (using the telephone, managing money, taking medications, shopping, preparing meals) at each interview. The ADL-IADL score was the sum of the dependencies for the combined 11 items. The year of and age at disability onset were estimated as the first year and age when a respondent’s ADL-IADL score exceeded 5.

Cognitive information is provided in the HRS as part of the core survey interviews. For self-respondents, cognitive impairment was determined using a performance-based measure, a modified version of the Telephone Interview for Cognitive Status, a validated cognitive screening instrument patterned on the Mini-Mental State Examination and specifically designed for population-based studies.^20^ For respondents unable to complete the interview, we used an 11-point scale composed of the proxy’s assessment of the respondent’s memory and IADL difficulties and the interviewer’s assessment of the respondent’s cognitive impairment.^7,21^ The year of and age at onset of cognitive impairment were defined as the first year and age when the respondent was assessed as having severe cognitive impairment.^22^ Death and the year of death were confirmed using the National Death Index and the Social Security Death Index. Fewer than 4.8% of values were missing among the diabetes-related outcomes in the HRS; only respondents with nonmissing information on diabetes and outcomes were used in the analysis.

#### Covariates

Demographic variables included age, sex, self-reported race and ethnicity, marital or partner status, educational attainment, wealth (net worth), and BMI. Self-reported race and ethnicity were classified as Hispanic, non-Hispanic Black, non-Hispanic White, and other (includes American Indian or Alaska Native, Asian, and Native Hawaiian or other Pacific Islander). Race and ethnicity represent one of the social determinants of health and so may be associated with the study’s outcomes. The study included other social determinants of health, including educational attainment and net worth. Comorbid conditions included self-reported lung disease, cancer, and arthritis. For respondents with diabetes, we defined 2 medication covariates, insulin use and oral medication use, using self-reported answers. Each covariate was classified into ever-user or nonuser categories.

### Statistical Analysis

Data were analyzed from June 1, 2021, to July 31, 2022. We performed descriptive analyses to characterize the age-at-diagnosis groups. Descriptive comparisons were performed using analysis of variance or a rank-sum test for continuous measures and the χ^2^ test for categorical measures.

For each outcome of interest except mortality, we first excluded the respondents having the outcome before diabetes diagnosis. We then identified a matched nondiabetes control group for each age-at-diagnosis group using a propensity score–matching technique on the calendar year of diabetes diagnosis to reduce the confounding effects of age and demographic and comorbidity characteristics. Propensity scores were constructed using logistic regression with diabetes (yes or never) as the dependent variable; the independent variables were age, sex, race and ethnicity, marital status, educational attainment, wealth, HRS birth cohort, calendar year of diabetes diagnosis, BMI, ADL-IADL score, cognitive status, and comorbid conditions present in the calendar year of diabetes diagnosis. Each matched control (MC) was required to be outcome free at the matched calendar year and was selected at a ratio of 1:1 by the nearest neighbor method measured by the generalized linear model distance.^23^ Controls for the 3 diabetes age-at-diagnosis groups were constructed separately and were mutually exclusive. We then compared the incidence rates and cumulative incidence curves of the outcomes between the cases with diabetes and their MCs during the follow-up years. The starting point of the follow-up years was the year of diabetes diagnosis for cases and the matched calendar year for the MCs. Therefore, the time scale of the cumulative incidence curve is the duration of diabetes for cases and matched aging for MCs. Proportional hazard models were then specified using year to outcome as the dependent variables; covariates included diabetes (yes or MC), age-at-diagnosis group, and their interaction terms. The interaction terms explicitly tested the hypothesis of whether the hazard ratios (HRs), which compare the respondents with diabetes vs their MCs on the outcomes, were the same among the age-at-diagnosis groups. Death was treated as a competing outcome for the outcomes of heart disease, stroke, disability, and cognitive impairment.^24^ Statistical significance was indicated at 2-sided *P* < .05.

To evaluate the effects of insulin and oral medication, we compared the respondents with diabetes and their MCs in the following subgroups: insulin and oral medication nonuser, oral medication user and insulin nonuser, and insulin user. We further extended the proportional hazard model using age at diagnosis as a continuous covariate. Sensitivity analyses were performed incorporating respondent-level weights (from the year of matching). Last, we performed sensitivity analyses by using 1:2 and 1:3 ratios in propensity score matching and by varying the disability cutoff at ADL-IADL scores (3-6) to ensure that there were no differences in overall conclusions or patterns. All analyses were performed using R, version 3.5.1 (R Program for Statistical Computing),packages MatchIt, compeit, and cmprsk.

## Results

Among the 39 093 HRS respondents 50 years and older across the 1995 to 2018 waves, 39 034 respondents had information on diabetes at their baseline interview (Figure 1). Of these, 7739 developed diabetes in the succeeding waves (4267 women [55.1%]; 3472 men [44.9%]; mean [SD] age at diagnosis, 67.42 [9.88] years; median follow-up, 20 [IQR, 8-26] years); 28 321 never reported having diabetes. The estimated diabetes incidence rate was 17.4 (95% CI, 17.0-17.8) per 1000 person-years. The age-at-diagnosis groups included 1866 respondents at 50 to 59 years; 2834, at 60 to 69 years; and 3039, at 70 years and older.

The Table presents the demographic and comorbidity characteristics at the year of diabetes diagnosis for each of the 3 age-at-diagnosis groups. Compared with the group aged 50 to 59 years at diagnosis, those 70 years and older at diagnosis were more likely to be male (1383 [45.5%] vs 721 [38.6%]), non-Hispanic White (2240 [73.7%] vs 930 [49.9%]), and not married (1433 [47.1%] vs 490 [26.3%]) and to have less educational attainment (>12 years, 867 [28.5%] vs 737 [39.7%]) and wealth (<$40 000, 2286 [75.2%] vs 981 [52.6%]). In addition, adults with an older age at diagnosis had a lower median BMI (27.4 [IQR, 24.3-30.9] vs 31.3 [IQR, 27.4-35.9]), less smoking (193 [6.4%] vs 372 [19.9%]), and higher prevalence of existing comorbid conditions (eg, heart disease, 1350 [44.4%] vs 387 [20.7%]) and disability (267 [8.8%] vs 36 [1.9%]). The demographic characteristics comparing the 7739 respondents with diabetes and the 28 321 respondents without diabetes by the year of diabetes diagnosis or baseline are summarized in eTable 1 in the Supplement.

**Table.** Characteristics of HRS Respondents With Diabetes by Age at Diagnosis and Overall

Characteristics	Age-at-diagnosis group^a^				*P* value^b^
	Overall (N = 7739)	50-59 y (n = 1866)	60-69 y (n = 2834)	≥70 y (n = 3039)	
Sex					
Women	4267 (55.1)	1145 (61.4)	1466 (51.7)	1656 (54.5)	<.001
Men	3472 (44.9)	721 (38.6)	1368 (48.3)	1383 (45.5)	
Race and ethnicity					
Hispanic	726 (9.4)	272 (14.6)	279 (9.9)	175 (5.8)	<.001
Non-Hispanic Black	1730 (22.4)	521 (28.0)	674 (23.8)	535 (17.6)	
Non-Hispanic White	4891 (63.3)	930 (49.9)	1721 (60.9)	2240 (73.7)	
Other^c^	383 (5.0)	140 (7.5)	154 (5.4)	89 (2.9)	
Educational attainment, y					
<12	2682 (34.8)	550 (29.6)	932 (33.0)	1200 (39.5)	<.001
12	2425 (31.4)	568 (30.6)	885 (31.3)	972 (32.0)	
>12	2610 (33.8)	737 (39.7)	1006 (35.6)	867 (28.5)	
Cohort^d^					
AHEAD	1394 (18.0)	0	0	1394 (45.9)	<.001
CODA	765 (9.9)	0	176 (6.2)	589 (19.4)	
HRS (refers to original HRS cohort)	2960 (38.2)	521 (27.9)	1536 (54.2)	903 (29.7)	
WB	850 (11.0)	311 (16.7)	399 (14.1)	140 (4.6)	
BB	1770 (22.9)	1034 (55.4)	723 (25.5)	13 (0.4)	
Age at diagnosis, mean (SD), y	67.4 (9.9)	55.5 (2.8)	64.2 (2.8)	77.7 (5.8)	<.001
Not married or living alone	2805 (36.2)	490 (26.3)	882 (31.1)	1433 (47.1)	<.001
Wealth, $					
<40 000	4963 (64.1)	981 (52.6)	1696 (59.8)	2286 (75.2)	<.001
40 000-149 999	2420 (31.3)	772 (41.4)	991 (35.0)	657 (21.6)	
150 000-299 999	280 (3.6)	93 (5.0)	113 (4.0)	74 (2.4)	
≥300 000	76 (1.0)	20 (1.1)	34 (1.2)	22 (0.7)	
BMI, median (IQR)	29.1 (25.7-33.0)	31.3 (27.4-35.9)	30.1 (27.1-34.0)	27.4 (24.3-30.9)	<.001
Current smoker	969 (12.5)	372 (19.9)	404 (14.3)	193 (6.4)	<.001
Comorbidity					
Heart disease	2584 (33.4)	387 (20.7)	847 (29.9)	1350 (44.4)	<.001
Stroke	901 (11.6)	125 (6.7)	260 (9.2)	516 (17.0)	<.001
Lung disease	1012 (13.1)	217 (11.6)	372 (13.1)	423 (13.9)	.07
Cancer	1076 (13.9)	129 (6.9)	334 (11.8)	613 (20.2)	<.001
Arthritis	4851 (62.7)	945 (50.6)	1833 (64.7)	2073 (68.2)	<.001
Disability	375 (4.8)	36 (1.9)	72 (2.5)	267 (8.8)	<.001
Cognitive impairment					
Normal	5579 (72.1)	1550 (83.1)	2209 (77.9)	1820 (59.9)	<.001
Minor impairment	1454 (18.8)	264 (14.1)	476 (16.8)	714 (23.5)	
Severe impairment	706 (9.1)	52 (2.8)	149 (5.3)	505 (16.6)	
During follow-up					
Insulin use	2183 (28.2)	701 (37.6)	773 (27.3)	709 (23.3)	<.001
Oral medication for diabetes	6209 (80.2)	1521 (81.5)	2361 (83.3)	2327 (76.6)	<.001
Death	3555 (45.9)	507 (27.2)	1093 (38.6)	1955 (64.3)	<.001
Follow-up, median (IQR), y	20 (8-26)	12 (8-20)	20 (12-26)	25 (20-26)	<.001

Abbreviations: AHEAD, Assets and Health Dynamics Among the Oldest Old; BB, Baby Boomer (combination of Early and Middle Baby Boomers); BMI, body mass index (calculated as weight in kilograms divided by height in meters squared); CODA, Children of the Depression; HRS, the Health and Retirement Study; WB, War Baby.

^a^ Unless indicated otherwise, data are expressed as No. (%) of participants. Percentages have been rounded and may not total 100. Owing to missing data, some numbers of participants may be less than the totals given in the column headings.

^b^ *P* values are from ANOVA or rank sum test (for BMI) for comparing continuous covariates and the χ^2^ test for comparing categorical covariates.

^c^ Includes American Indian or Alaska Native, Asian, and Native Hawaiian or other Pacific Islander.

^d^ Includes 6 birth cohorts: the AHEAD cohort (individuals born before 1924), the CODA cohort (individuals born between 1924 and 1930), the original HRS cohort (individuals born between 1931 and 1941), the WB cohort (individuals born between 1942 and 1947), the Early Baby Boomer cohort (individuals born between 1948 and 1953), and the Middle Baby Boomer cohort (individuals born between 1954 and 1959).

A propensity score–matched control respondent was selected for each respondent with diabetes at that respondent’s year of diabetes diagnosis, using the variables in the Table. The characteristics of the diabetes and MC groups after propensity score matching are presented in eTable 2 in the Supplement.

**Figure 2.**
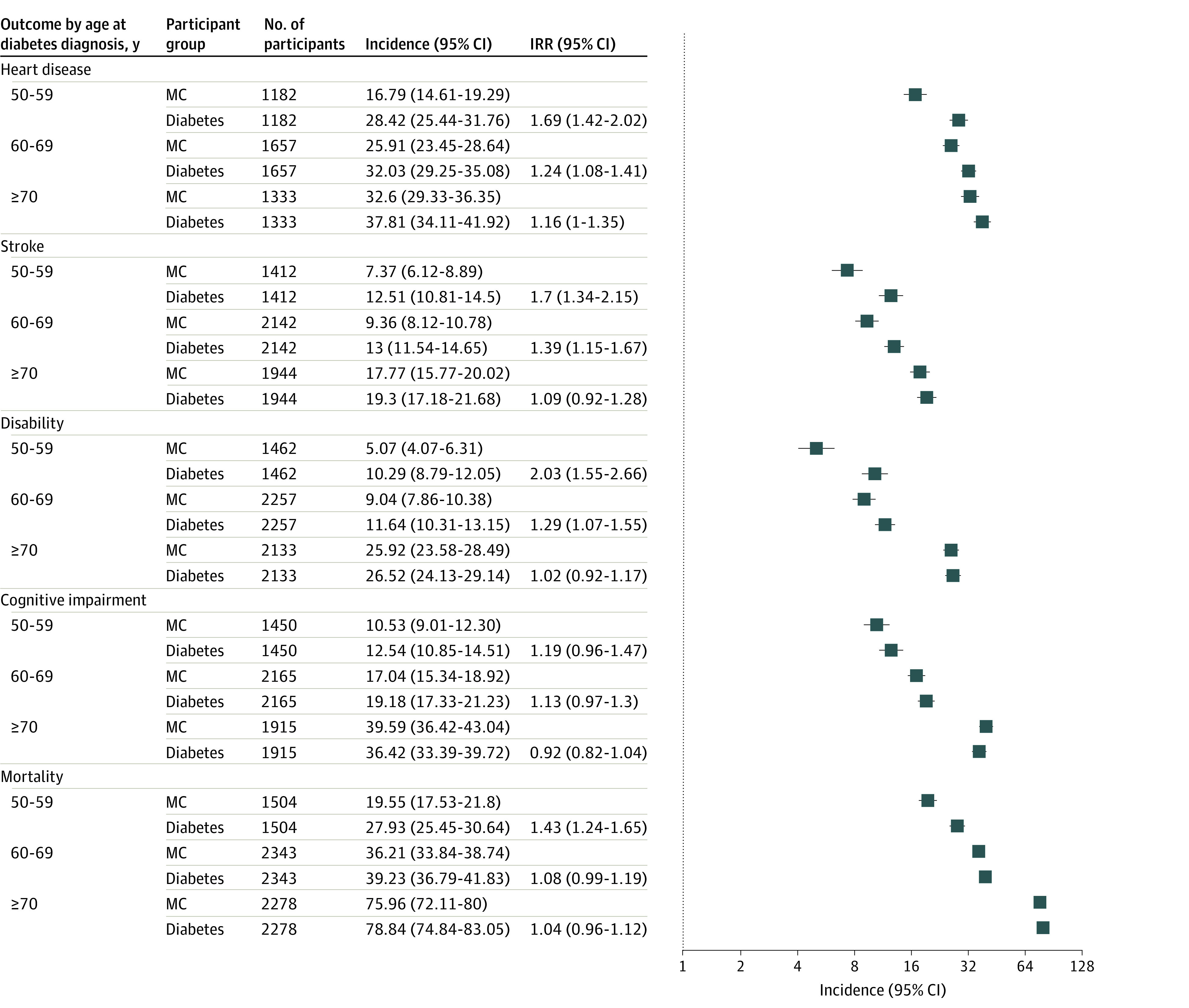
Incidence of Distal Outcomes for Each Age-at-Diagnosis Group With Diabetes and Its Matched Control (MC) Group Incidence per 1000 person-years is listed for heart disease, stroke, disability (activities of daily living and instrumental activities of daily living score >5), severe cognitive impairment, and all-cause mortality for each age-at-diagnosis group with diabetes and its MC group. Incidence rate ratios (IRRs) between participants with diabetes and the MCs for each outcome are estimated for each age-at-diagnosis group separately.

Figure 2 presents the incidence of heart disease, stroke, disability, and cognitive impairment and mortality for each diabetes age-at-diagnosis group and its corresponding MC group; it also presents the incident rate ratio (IRR) between the diabetes and MC groups. For all outcomes, although the incidences increased with aging, the IRRs between each diabetes group and its MC group decreased with advancing age at diagnosis of diabetes. For example, with advancing age at diabetes diagnosis compared with each MC group, the incidence rates of heart disease were 28.42 (95% CI, 25.44-31.76) vs 16.79 (95% CI, 14.61-19.29) per 1000 person-years for 50 to 59 years of age; 32.03 (95% CI, 29.25-35.08) vs 25.91 (95% CI, 23.45-28.64) per 1000 person-years for 60 to 69 years of age; and 37.81 (95% CI, 34.11-41.92) vs 32.60 (95% CI, 29.23-36.35) per 1000 person-years for 70 years or older. However, the IRRs were 1.69 (95% CI, 1.42-2.02) for those aged 50 to 59 years at diabetes diagnosis, 1.24 (95% CI, 1.08-1.41) for those aged 60 to 69 years at diabetes diagnosis, and 1.16 (95% CI, 1.00-1.35) for those 70 years and older at diagnosis. A similar pattern was observed for the other outcomes. Specifically, respondents who were diagnosed at age 50 to 59 years had a significantly higher incidence of stroke (IRR, 1.70 [95% CI, 1.34-2.15]), disability (IRR, 2.03 [95% CI, 1.55-2.66]), and mortality (IRR, 1.43 [95% CI, 1.24-1.65]) than their MCs. Respondents who were diagnosed at age 60 to 69 years had reduced but significantly higher incidences of stroke (IRR, 1.39 [95% CI, 1.15-1.67]) and disability (IRR, 1.29 [95% CI, 1.07-1.55]) than their MCs. Respondents who were diagnosed at age 70 years or older did not have any outcome incidences that were significantly higher than those of their MCs.

**Figure 3.**
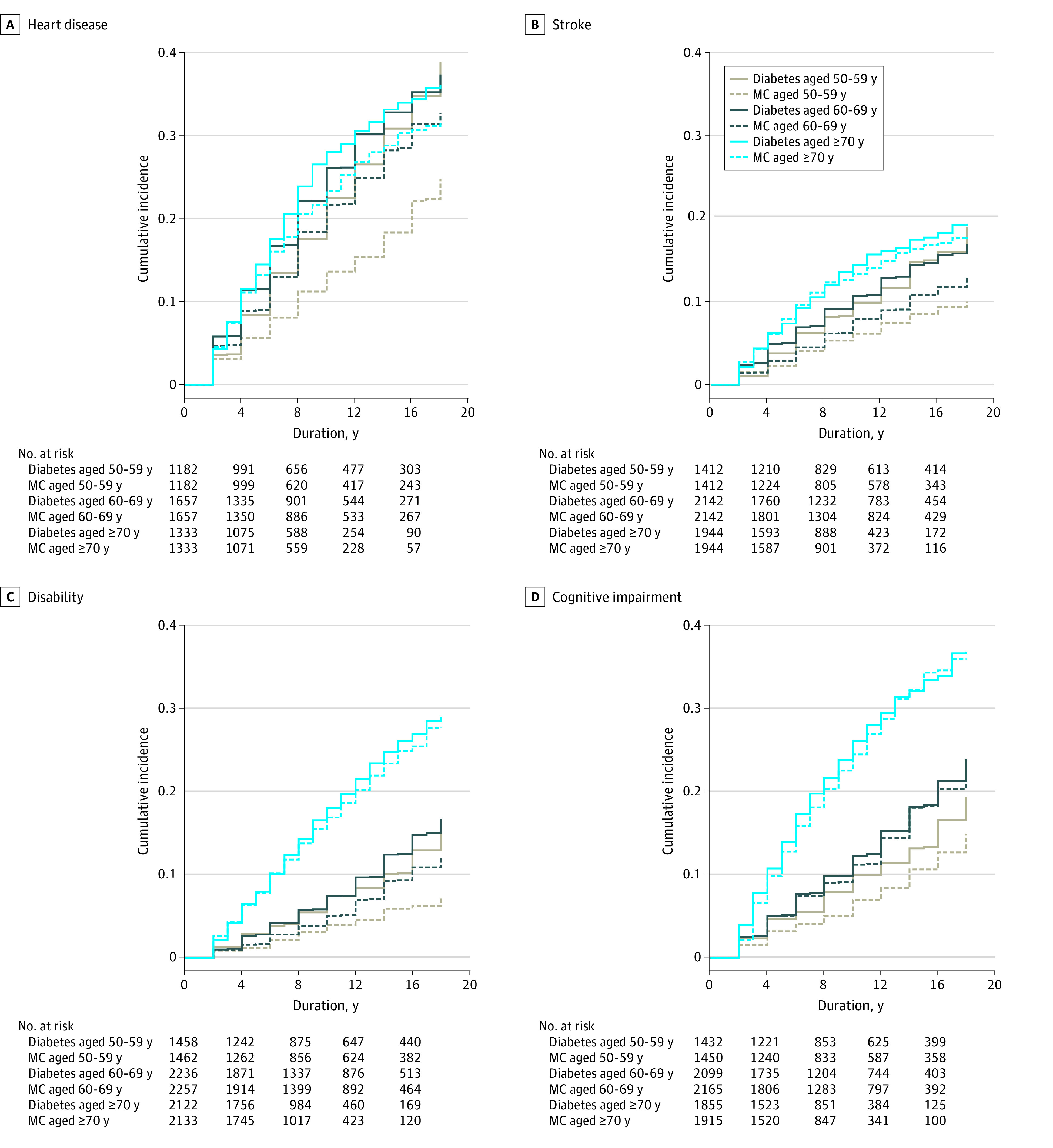
Cumulative Incidence Curves of Distal Outcomes for Each Age-at-Diagnosis Group With Diabetes and Its Matched Control (MC) Group Disability is indicated by activities of daily living and instrumental activities of daily living score of greater than 5. The x-axis of each figure is the duration of diabetes for participants with diabetes and aging for the MCs. The starting point of the follow-up years is the year of diabetes diagnosis for participants with diabetes and the matched calendar year for the MCs.

The cumulative incidence curves of the 4 diabetes-related outcomes for each age-at-diagnosis group and its MC group are shown in Figure 3. The cumulative incidence curve of mortality is shown in eFigure 1 in the Supplement. Consistent with the observation of higher incidence with aging (Figure 2), the groups with a diagnosis at 70 years and older and their MC groups had the highest cumulative incidence for all outcomes. The groups with a diagnosis at age 60 to 69 years and their MC groups had an intermediate cumulative incidence, whereas the groups with a diagnosis at 50 to 59 years of age and their MC groups had the lowest cumulative incidence. However, the differences in the trajectories of cumulative incidence between the diabetes and the respective MC groups were greatest in the groups aged 50 to 59 years, intermediate in the groups aged 60 to 69 years, and almost the same for the groups aged 70 years and older. These findings are consistent with the reduced IRRs with advancing age at diabetes diagnosis in Figure 2. A significant departure was seen for the curves of groups 70 years and older (both diabetes and MC) away from those of the other 2 age-at-diagnosis groups, especially for disability and cognitive impairment, underscoring their increased risk due to aging but not diabetes.

**Figure 4.**
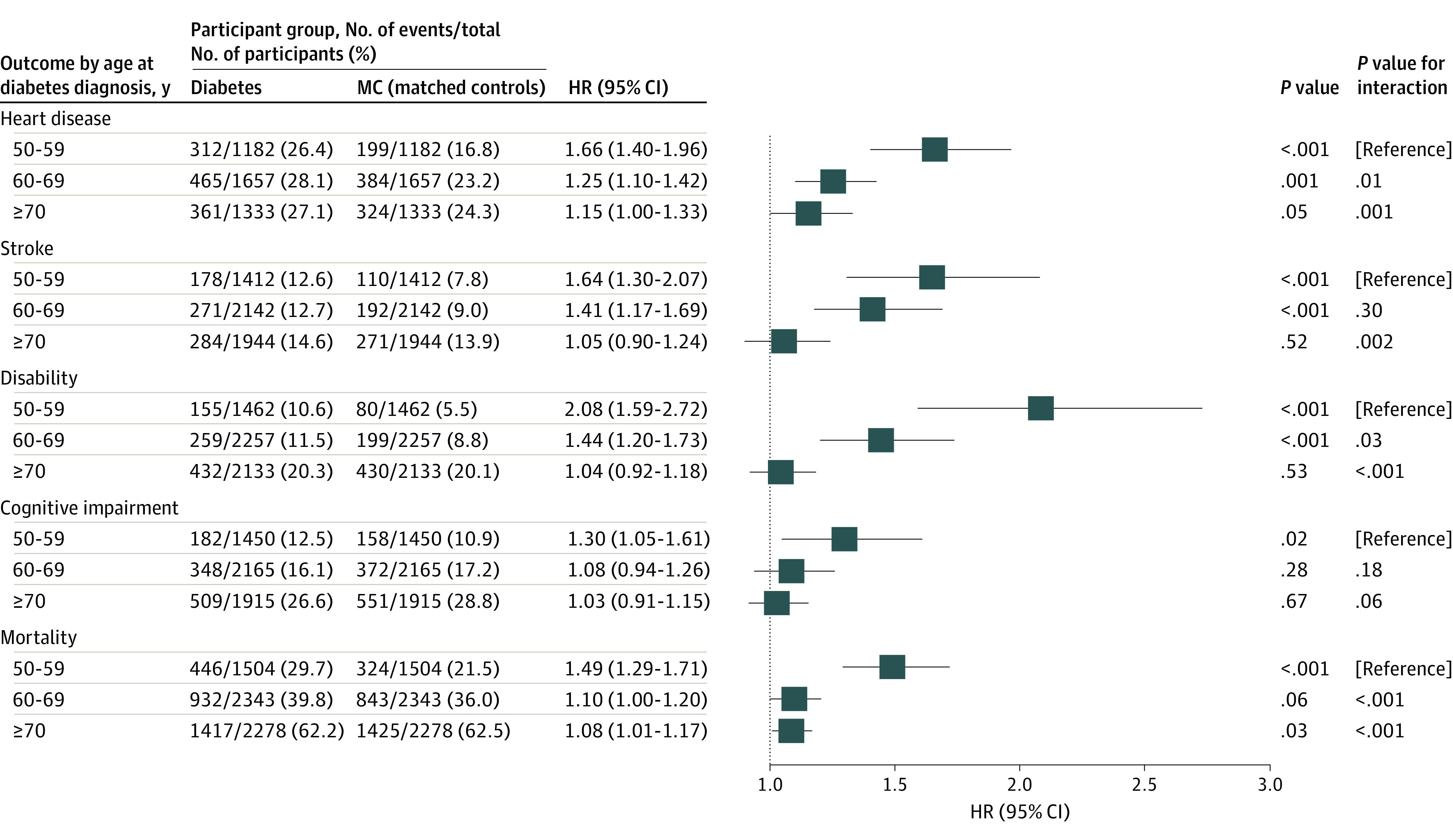
Hazard Ratios (HRs) of Diabetes on Distal Outcomes Compared With Matched Controls (MCs) by Diabetes Age-at-Diagnosis Groups The HRs between diabetes age-at-diagnosis and MC groups are estimated for each outcome. The *P* value column tests the corresponding HR of diabetes to the null hypothesis (HR of 1.00). The interaction *P* value column compares the HR of diabetes of the corresponding age-at-diagnosis group with the HR of diabetes in the reference group with age at diagnosis at 50 to 59 years.

Figure 4 depicts the HRs of diabetes on the distal outcomes by age-at-diagnosis groups. Diabetes was associated with elevated risks of heart disease (HR, 1.66 [95% CI, 1.40-1.96]), stroke (1.64 [95% CI, 1.30-2.07]), disability (HR, 2.08 [95% CI, 1.59-2.72]), cognitive impairment (HR, 1.30 [95% CI, 1.05-1.61]), and mortality (HR, 1.49 [95% CI, 1.29-1.71]) for the groups aged 50 to 59 years compared with their MC groups but was associated only with elevated mortality for the group aged 70 years and older (HR 1.08 [95% CI, 1.01-1.17]). For all outcomes except cognitive impairment, the HRs of respondents with diabetes vs their MCs were significantly lower in the groups aged 70 years and older than those in the groups aged 50 to 59 years (all *P* < .05 for interaction). The HRs of respondents with diabetes vs MCs for the groups aged 60 to 69 years were significantly lower than the HRs for the groups aged 50 to 59 years for heart disease (HR, 1.25 [95% CI, 1.10-1.42]), disability (HR, 1.44 [95% CI, 1.20-1.73]), and all-cause mortality (HR, 1.10 [95% CI, 1.00-1.20]) (all *P* < .05 for interaction).

The results incorporating respondent-level weights at the year of matching are shown in eFigure 2 in the Supplement. The overall trend for age at diagnosis remained the same except for cognitive impairment, for which no significant associations were found for any age-at-diagnosis groups. The decreasing trend persisted when age at diagnosis was used as a continuous variable (eFigure 4 in the Supplement).

eFigure 3 in the Supplement summarizes the association of diabetes with the distal outcomes when stratified by insulin and oral medication use. These findings are consistent with our primary results for insulin users and oral medication users. For the smallest subgroup of respondents with diabetes—those who do not use insulin or an oral medication—the association of diabetes with distal outcomes appeared to persist across all age-at-diagnosis groups, although the analyses were underpowered owing to limited sample size.

## Discussion

In this cohort study using 23 years of longitudinal data for adults 50 years and older drawn from a national health interview survey, we provide evidence of the differential association of diabetes with incident distal outcomes across varying ages at diabetes diagnosis while accounting for diabetes duration. Our use of an MC group helped us separate the association of diabetes with these outcomes from the association with increasing age.

Emerging evidence^25,26,27^ suggests that earlier diagnosis of diabetes is associated with an increased risk of cardiovascular health outcomes and mortality, compared with later diagnosis. An Australian national prospective study found that age at diabetes diagnosis was inversely associated with the risk of all-cause mortality, especially cardiovascular mortality.^25^ Similar findings were observed in the Swedish National Diabetes Registry^26^ and a cross-sectional analysis of Chinese patients with diabetes.^27^ Although several studies have demonstrated increased risk for cognitive impairment^28,29,30^ and disability^31,32,33^ in older adults with type 2 diabetes, none have examined the effect of age at diabetes diagnosis on these outcomes. The findings of our study suggest that these risks are predominantly present in individuals who receive a diabetes diagnosis at a younger age.

The mechanisms that link earlier diabetes diagnosis to worse outcomes are not completely understood. The longer duration of diabetes in individuals with earlier diagnosis has been reported in several previous studies.^25,27^ In contrast, in our study, the age-at-diagnosis association persisted when diabetes duration was fully adjusted by comparing incident outcomes starting from the age at diabetes diagnosis. Another plausible explanation is that adults who are younger at diagnosis may have a more physiologically aggressive form of diabetes, with worse glycemic control, beta cell dysfunction, insulin insufficiency, and insulin resistance. This explanation is supported by a cross-sectional study from National Health and Nutrition Examination Survey, which found that adults with earlier diagnosis of diabetes (<65 years) had significantly worse glycemic control than those with a later diagnosis (≥65 years).^34^ Vascular dysfunction, poor glycemic control, and insulin resistance are also risk factors for cognitive impairment^35^ and worse physical functioning.^36^

### Strengths and Limitations

Compared with other studies, ours has several unique advantages. First, we differentiated age, age at diagnosis, and diabetes duration. Comparing incident outcomes starting from the year of diabetes diagnosis for the respondents with diabetes (duration) and the outcomes during aging for the MC groups allows for the separation of associations between age at diagnosis and duration and between diabetes and aging.^25,37,38,39,40,41,42^ The use of the HRS, with multiple variables collected longitudinally for respondents of all age-at-diagnosis groups during 2 decades, enables us to place diabetes diagnosis, prevalent comorbid conditions at the time of diabetes diagnosis, and incident comorbid outcomes after diabetes diagnosis along a time continuum into the oldest respondents. Lifestyle and behavioral effects and treatment effects have undergone secular change during the 2 decades of the study, which underscores the importance of matching respondents with diabetes to the no-diabetes control respondents of the same age from the same birth cohort in the same calendar year. Thus, we provide powerful evidence of heterogeneous trajectories of distal outcomes in people with diabetes at varying ages of diagnosis.

Our study also has limitations. We cannot rule out potential measurement errors due to the self-report of diabetes, its distal outcomes (heart disease, stroke, disability), and certain covariates. Self-report cannot distinguish between type 1 and type 2 diabetes, although self-reported diabetes is often used for diabetes ascertainment in national studies, and most incident diabetes cases at age 50 years and older are type 2 diabetes. The identified MCs may be subject to ascertainment bias because they may have shorter follow-up to report diabetes. Last, this study does not explore the potential impact of prediabetes, which is increasingly understood as not only a risk for development of type 2 diabetes, but also for poorer health outcomes.

## Conclusions

This cohort study provides evidence of the differential association of diabetes with incident distal outcomes across varying ages at diabetes diagnosis while accounting for diabetes duration. With increasing age at diagnosis, diabetes is associated with significantly decreased risks of incident comorbid outcomes. In adults who were younger at diabetes diagnosis (50-59 years), diabetes was significantly associated with an increased HR for each distal outcome. In contrast, the association was not significant for the oldest diagnosis group (≥70 years). We believe this study provides a framework for future studies that can explore other key variables associated with diabetes-related health outcomes. Metabolic mechanisms, lifestyle and behaviors, social determinants of health, and diabetes management all affect age at diabetes diagnosis and aging with diabetes. Future studies should address the full complexity of diabetes and its effects over time.

## Notes

Supplement. **eMethods.** Adjudication Methodology **eFigure 1.** The Cumulative Incidence Curves of All-Cause Mortality for Each Diabetes Age-at-Diagnosis Group and Its Matched-Control Group **eFigure 2.** The Hazard Ratios of Diabetes on Distal Outcomes Incorporating Respondent-Level Sampling Weights at the Year of Matching **eFigure 3.** The Stratified Analyses of Diabetes on Distal Outcomes Based on Insulin Use and Oral Medication Use **eFigure 4.** The Hazard Ratios of Diabetes on Distal Outcomes Against Continuous Age-at-Diagnosis **eTable 1.** Demographic Characteristics of Diabetes and Diabetes-Free Participants at the Year of Diabetes/Baseline **eTable 2.** Demographic Characteristics of Each Diabetes Age-at-Diagnosis Group and Its Matched Control Group at the Year of Diabetes Diagnosis/Matching
