# Supplementary material for: Associations of Age at Diagnosis and Duration of Diabetes With Morbidity and Mortality Among Older Adults
Source: JAMA Netw Open. 2022 Sep 30;5(9):e2232766. doi: 10.1001/jamanetworkopen.2022.32766 (PMC9526092; doi:10.1001/jamanetworkopen.2022.32766)
Supplement: Supplement. — eMethods. Adjudication Methodology eFigure 1. The Cumulative Incidence Curves of All-Cause Mortality for Each Diabetes Age-at-Diagnosis Group and Its Matched-Control Group eFigure 2. The Hazard Ratios of Diabetes on Distal Outcomes Incorporating Respondent-Level Sampling Weights at the Year of Matching eFigure 3. The Stratified Analyses of Diabetes on Distal Outcomes Based on Insulin Use and Oral Medication Use eFigure 4. The Hazard Ratios of Diabetes on Distal Outcomes Against Continuous Age-at-Diagnosis eTable 1. Demographic Characteristics of Diabetes and Diabetes-Free Participants at the Year of Diabetes/Baseline eTable 2. Demographic Characteristics of Each Diabetes Age-at-Diagnosis Group and Its Matched Control Group at the Year of Diabetes Diagnosis/Matching [file jamanetwopen-e2232766-s001.pdf]

Supplementary Online Content

Cigolle CT, Blaum CS, Lyu C, Ha J, Kabeto M, Zhong J. Associations of age at diagnosis and duration of diabetes with morbidity and mortality among older adults. *JAMA Netw Open*. 2022;5(9):e2232766. doi:10.1001/jamanetworkopen.2022.32766

eMethods. Adjudication Methodology
eFigure 1. The Cumulative Incidence Curves of All-Cause Mortality for Each Diabetes Age-at-Diagnosis Group and Its Matched-Control Group
eFigure 2. The Hazard Ratios of Diabetes on Distal Outcomes Incorporating Respondent-Level Sampling Weights at the Year of Matching
eFigure 3. The Stratified Analyses of Diabetes on Distal Outcomes Based on Insulin Use and Oral Medication Use
eFigure 4. The Hazard Ratios of Diabetes on Distal Outcomes Against Continuous Age-at-Diagnosis
eTable 1. Demographic Characteristics of Diabetes and Diabetes-Free Participants at the Year of Diabetes/Baseline
eTable 2. Demographic Characteristics of Each Diabetes Age-at-Diagnosis Group and Its Matched Control Group at the Year of Diabetes Diagnosis/Matching

This supplementary material has been provided by the authors to give readers additional information about their work.

© 2022 Cigolle CT et al. *JAMA Network Open*.

eMethods.

Adjudication

Methodology

The description of the adjudication methodology is taken from:

Cigolle CT, Nagel CL, Blaum CS, Liang J, Quinones AR. Inconsistency in the Self-report of Chronic Diseases in Panel Surveys: Developing an Adjudication Method for the Health and Retirement Study. *J Gerontol B Psychol Sci Soc Sci*. Jun 14 2018;73(5):901-912. doi:10.1093/geronb/gbw063

Specifically, these paragraphs are taken from pages 901-902.

"In its biennial core survey interviews, the HRS includes follow-up questions about each chronic disease that are answered by all respondents who answered "Yes" to having the disease in that wave (see Variables and Their Measurement: Chronic Diseases). We used the responses to these follow-up questions to form algorithms to adjudicate inconsistencies in the self- reported disease data across HRS waves. For example, if a respondent ever reported having heart surgery, he is deemed to always have heart disease, even in waves where he denied having heart disease. Similarly, if a respondent ever reported using insulin for diabetes, he is deemed to always have diabetes, even in waves where he denied having diabetes.

"We developed levels of adjudication that address each type of inconsistency in respondents' self-report of chronic diseases:

© 2022 Cigolle CT et al. *JAMA Network Open*.

- Adjudication Level One: If the respondent disputed the prior wave record but now replies that he has the disease, we adjudicated his response as “Yes”. If the respondent disputed the prior wave record and replies that he does not now have the disease, we examined his prior wave responses for evidence of having the disease. If evidence was present, we adjudicated his response as “Yes”; if evidence was not present, we adjudicated his response as “No”.
- Adjudication Level Two: For “Don’t know” and “Refuse to answer” responses, we examined the respondent’s prior wave responses for evidence of having the disease. If evidence was present, we adjudicated his response as “Yes”; if evidence was not present, we adjudicated his response as “No”.
- Adjudication Level Three: For a “No” response in the current wave being preceded by a “Yes” response in the prior wave, we examined the respondent’s prior wave responses for evidence of having the disease. If evidence was present, we adjudicated his response as “Yes”; if evidence was not present, we adjudicated his response as “No”.
- Adjudication Level Four: When a “Yes” response in the current wave was followed by a dispute of this “Yes” response in the succeeding wave, we examined the respondent’s current wave responses for evidence of having the disease. If evidence was present, we adjudicated his response as “Yes”; if evidence was not present, we adjudicated his response as “No”.

Adjudication Levels 1 through 4 are conservative correctives, based on the logic of and the phrasing within the HRS questionnaires themselves. (Fisher, Faul, Weir, et al., 2005) Levels 1 through 4 address inconsistencies in chronic disease

© 2022 Cigolle CT et al. *JAMA Network Open*.

reporting by respondents between adjacent waves (that the respondent participated in). To restate, Levels 1 through 4 draw on evidence from the same wave or the immediately preceding wave."

For the present study, the evidence for diabetes includes (1) the self-reported answer "Yes" to at least one of the following questions and/or (2) A1c measurement ≥6.5.

- In order to treat or control your diabetes, are you now taking medication that you swallow?
- Are you now using insulin shots or a pump?

© 2022 Cigolle CT et al. *JAMA Network Open*.

eFigure 1. The Cumulative Incidence Curves of All-Cause Mortality for Each Diabetes Age-at-Diagnosis Group and Its Matched-Control Group

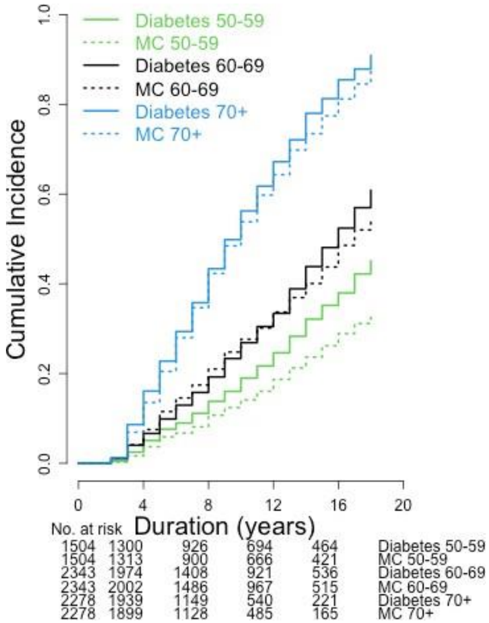

The starting point of the follow up years is the year of diabetes diagnosis for diabetes and the matched calendar year for the matched controls (MCs). Therefore, the time scale of the cumulative incidence curve is the duration of diabetes for diabetes patients and matched aging for MCs.

© 2022 Cigolle CT et al. *JAMA Network Open*.

eFigure 2. The Hazard Ratios of Diabetes on Distal Outcomes Incorporating Respondent-Level Sampling Weights at the Year of Matching

Outcome<br>Diabetes age-of-diagnosis	Events/N(%)<br>Diabetes	Events/N(%)<br>MC (Matched controls)	HR<br>95% CI	P value	Interaction P
Heart Disease					
50-59	312/1182(26)	199/1182(17)	1.53(1.22-1.92)	<0.001	ref
60-69	465/1657(28)	384/1657(23)	1.34(1.14-1.57)	<0.001	0.34
70+	361/1333(27)	324/1333(24)	1.07(0.91-1.26)	0.42	0.01
Stroke					
50-59	178/1412(13)	110/1412(8)	1.48(1.08-2.01)	0.01	ref
60-69	271/2142(13)	192/2142(9)	1.25(0.99-1.58)	0.06	0.41
70+	284/1944(15)	271/1944(14)	1.05(0.88-1.26)	0.56	0.06
Disability					
50-59	155/1462(11)	80/1462(5)	1.86(1.31-2.65)	0.001	ref
60-69	259/2257(11)	199/2257(9)	1.31(1.05-1.64)	0.02	0.1
70+	432/2133(20)	430/2133(20)	1.04(0.91-1.21)	0.55	0.003
Cognitive Impairment					
50-59	182/1450(13)	158/1450(11)	1.09(0.83-1.44)	0.54	ref
60-69	348/2165(16)	372/2165(17)	0.97(0.81-1.16)	0.75	0.49
70+	509/1915(27)	551/1915(29)	1.01(0.89-1.16)	0.83	0.64
Mortality					
50-59	446/1504(30)	324/1504(22)	1.28(1.06-1.55)	0.01	ref
60-69	932/2343(40)	843/2343(36)	1.15(1.02-1.29)	0.02	0.34
70+	1417/2278(62)	1425/2278(63)	1.08(0.99-1.17)	0.07	0.11

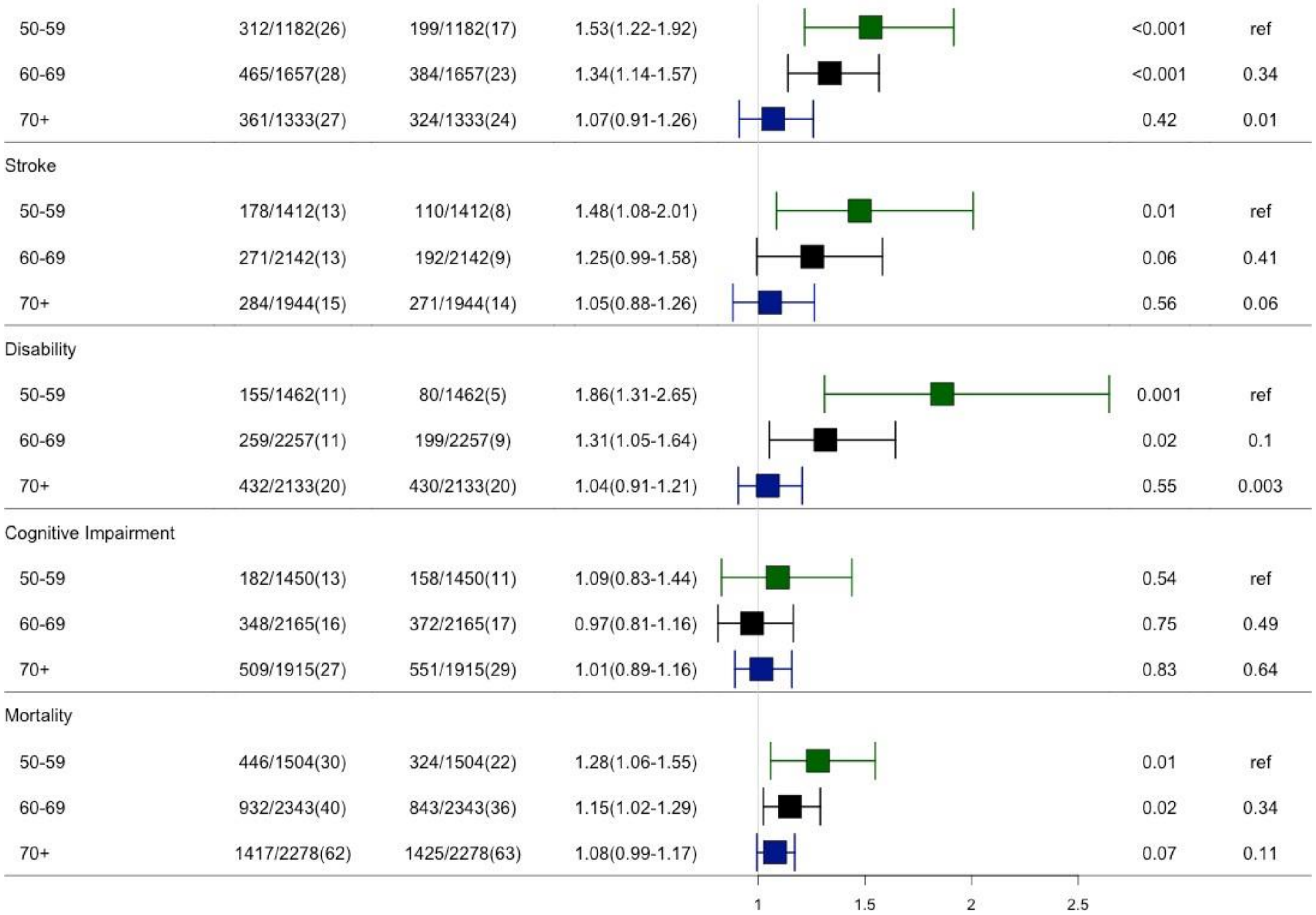

© 2022 Cigolle CT et al. *JAMA Network Open*.

The Events/N (%) denotes the number of events of each outcome by the total number of participants, who are outcome free at diabetes diagnosis or matched calendar year, (percentage) in either diabetes or matched controls (MCs) stratified by three diabetes age-of-diagnosis groups. The total Ns are the same between the diabetes and MCs as they were designed by 1-1 propensity score matching. The hazard ratios (95% confident interval) between diabetes and MCs are estimated for each outcome and each age-of-diagnosis group incorporating respondent-level weights at the year of matching. For participants who had 0 or missing weights at the year of matching, the average sample weights across all year were used. Participants who had 0 or missing weights throughout were removed in the Cox models. The p-value column presents the p-values testing the corresponding HR of diabetes to the null hypothesis (HR=1). The interaction p-value column presents the p-value comparing the HR of diabetes of the corresponding age of diagnosis group to the HR of diabetes in the reference age of diagnosis group 50-59.

© 2022 Cigolle CT et al. *JAMA Network Open*.

eFigure 3. The Stratified Analyses of Diabetes on Distal Outcomes Based on Insulin Use and Oral Medication Use

A. Insulin non-user and oral medication non-user

Outcome<br>Diabetes age-of-diagnosis	Events/N(%)<br>Diabetes	Events/N(%)<br>MC (Matched controls)	HR<br>95% CI	P value	Interaction P
Heart Disease					
50-59	18/77(23)	13/77(17)	1.81(0.91-3.61)	0.09	ref
60-69	49/132(37)	27/132(20)	2.42(1.57-3.75)	<0.001	0.49
70+	56/152(37)	44/152(29)	1.48(1.02-2.15)	0.04	0.61
Stroke					
50-59	10/97(10)	7/97(7)	1.75(0.68-4.51)	0.25	ref
60-69	25/137(18)	7/137(5)	4.13(1.82-9.37)	0.001	0.18
70+	34/197(17)	22/197(11)	1.72(1.01-2.91)	0.04	0.98
Disability					
50-59	7/91(8)	2/91(2)	4.7(1-22.11)	0.05	ref
60-69	13/140(9)	10/140(7)	1.46(0.65-3.29)	0.36	0.19
70+	32/202(16)	29/202(14)	1.33(0.82-2.17)	0.25	0.13
Cognitive Impairment					
50-59	13/98(13)	11/98(11)	2.03(0.89-4.61)	0.09	ref
60-69	33/155(21)	19/155(12)	2.29(1.3-4.04)	0.004	0.81
70+	60/207(29)	55/207(27)	1.52(1.06-2.17)	0.02	0.53
Mortality					
50-59	9/90(10)	9/90(10)	1.87(0.74-4.73)	0.19	ref
60-69	54/138(39)	35/138(25)	2.63(1.7-4.06)	<0.001	0.51
70+	110/202(54)	93/202(46)	1.69(1.28-2.23)	<0.001	0.84

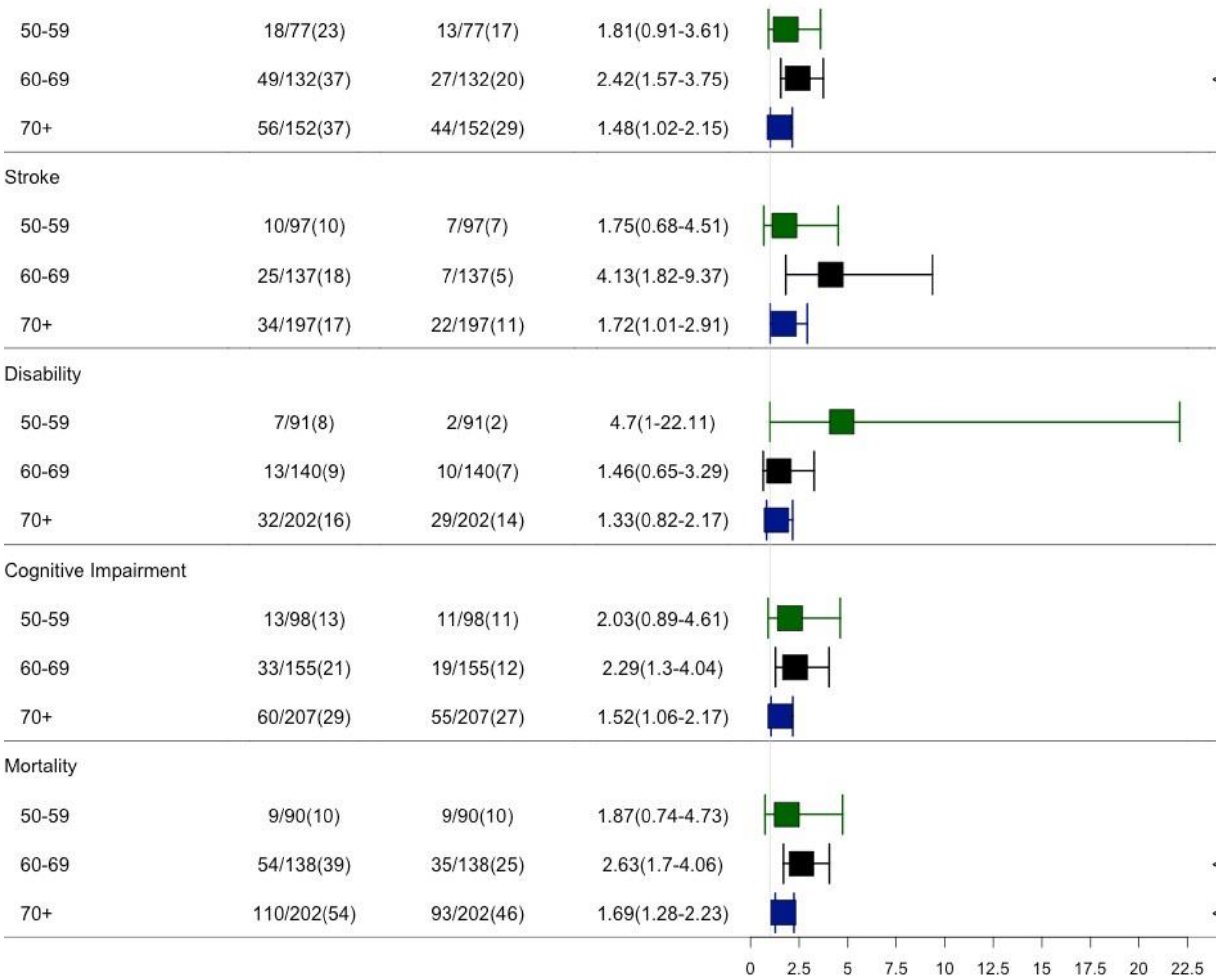

© 2022 Cigolle CT et al. *JAMA Network Open*.

B. Insulin user

Outcome<br>Diabetes age-of-diagnosis	Events/N(%)<br>Diabetes	Events/N(%)<br>MC (Matched controls)	HR<br>95% CI	P value	Interaction P
Heart Disease					
50-59	131/419(31)	93/419(22)	1.43(1.11-1.83)	0.005	ref
60-69	118/374(32)	120/374(32)	0.92(0.72-1.17)	0.51	0.01
70+	68/263(26)	65/263(25)	1.02(0.74-1.4)	0.92	0.1
Stroke					
50-59	89/559(16)	64/559(11)	1.33(0.98-1.81)	0.07	ref
60-69	90/583(15)	76/583(13)	1.13(0.84-1.52)	0.43	0.45
70+	71/433(16)	81/433(19)	0.83(0.61-1.13)	0.24	0.04
Disability					
50-59	91/604(15)	57/604(9)	1.65(1.19-2.3)	0.003	ref
60-69	118/625(19)	71/625(11)	1.71(1.29-2.28)	<0.001	0.88
70+	119/446(27)	119/446(27)	0.99(0.78-1.25)	0.94	0.01
Cognitive Impairment					
50-59	92/588(16)	77/588(13)	1.19(0.88-1.61)	0.25	ref
60-69	121/583(21)	126/583(22)	0.96(0.75-1.23)	0.76	0.28
70+	121/389(31)	158/389(41)	0.71(0.57-0.88)	0.002	0.006
Mortality					
50-59	269/645(42)	195/645(30)	1.4(1.17-1.69)	<0.001	ref
60-69	417/709(59)	368/709(52)	1.06(0.92-1.22)	0.39	0.02
70+	481/565(85)	458/565(81)	1.07(0.95-1.22)	0.27	0.02

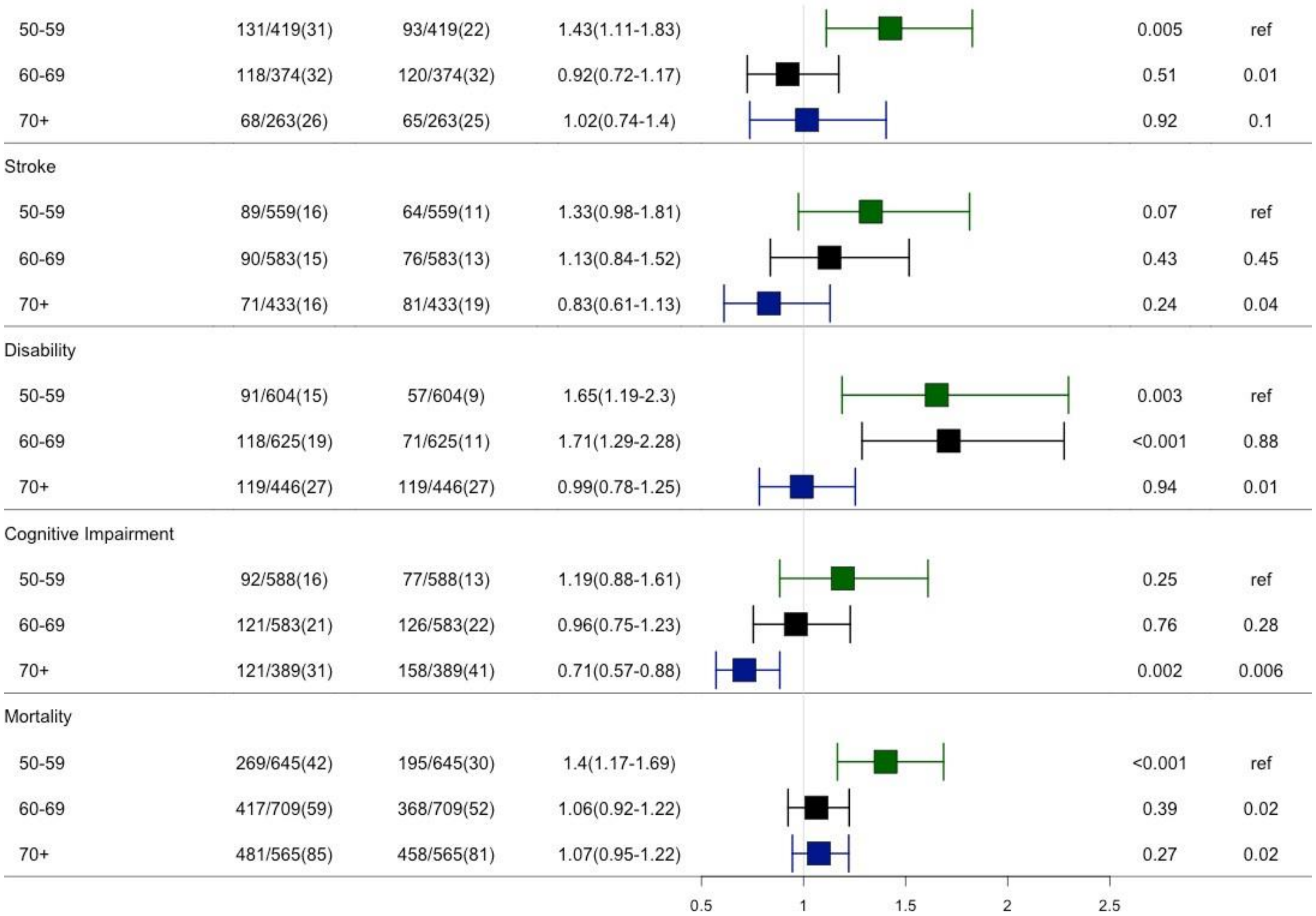

© 2022 Cigolle CT et al. *JAMA Network Open*.

C. Insulin non-user and oral medication user

Outcome / Diabetes age-of-diagnosis	Events/N(%) Diabetes	Events/N(%) MC (Matched controls)	HR 95% CI	P value	Interaction P
Heart Disease					
50-59	161/657(25)	93/657(14)	1.84(1.44-2.36)	<0.001	ref
60-69	298/1101(27)	235/1101(21)	1.33(1.12-1.56)	0.001	0.03
70+	236/888(27)	213/888(24)	1.15(0.96-1.37)	0.12	0.002
Stroke					
50-59	79/723(11)	38/723(5)	2.18(1.49-3.19)	<0.001	ref
60-69	155/1362(11)	108/1362(8)	1.45(1.14-1.84)	0.003	0.08
70+	178/1271(14)	168/1271(13)	1.07(0.87-1.32)	0.5	0.001
Disability					
50-59	57/733(8)	19/733(3)	3.09(1.85-5.16)	<0.001	ref
60-69	128/1427(9)	118/1427(8)	1.27(0.99-1.64)	0.06	0.002
70+	279/1437(19)	280/1437(19)	1.03(0.88-1.21)	0.68	<0.001
Cognitive Impairment					
50-59	77/730(11)	70/730(10)	1.35(0.97-1.88)	0.07	ref
60-69	194/1363(14)	222/1363(16)	1.07(0.88-1.3)	0.5	0.23
70+	328/1277(26)	336/1277(26)	1.12(0.97-1.3)	0.13	0.31
Mortality					
50-59	168/734(23)	120/734(16)	1.57(1.25-1.99)	<0.001	ref
60-69	461/1431(32)	440/1431(31)	1.04(0.91-1.19)	0.54	0.003
70+	825/1462(56)	874/1462(60)	1.03(0.93-1.13)	0.6	0.001

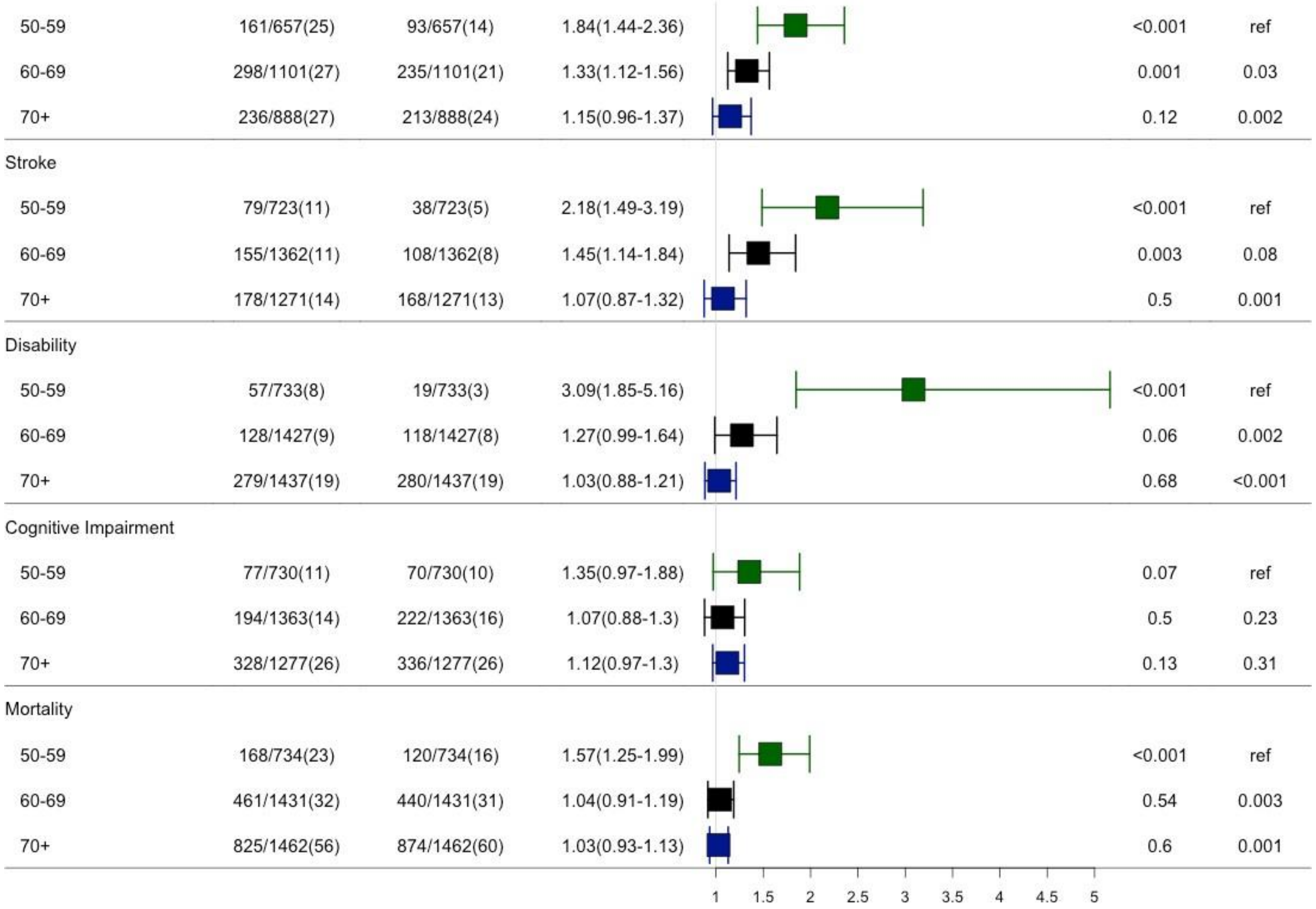

© 2022 Cigolle CT et al. *JAMA Network Open*.

The figures are separately for A) insulin non-user and oral medication non-user; B) insulin user; C) insulin non-user and oral medication user. The Events/N (%) denotes the number of events of each outcome by the total number of participants, who are outcome free at diabetes diagnosis or matched calendar year, (percentage) in either diabetes or matched controls (MCs) stratified by three diabetes age-of-diagnosis groups. The total Ns are the same between the diabetes and MCs as they were designed by 1-1 propensity score matching. The hazard ratios (95% confident interval) between diabetes and MCs are estimated for each outcome and each age-of-diagnosis group. The p-value column presents the p-values testing the corresponding HR of diabetes to the null hypothesis (HR=1). The interaction p-value column presents the p-value comparing the HR of diabetes of the corresponding age of diagnosis group to the HR of diabetes in the reference age of diagnosis group 50-59.

© 2022 Cigolle CT et al. *JAMA Network Open*.

eFigure 4. The Hazard Ratios of Diabetes on Distal Outcomes Against Continuous Age-at-Diagnosis

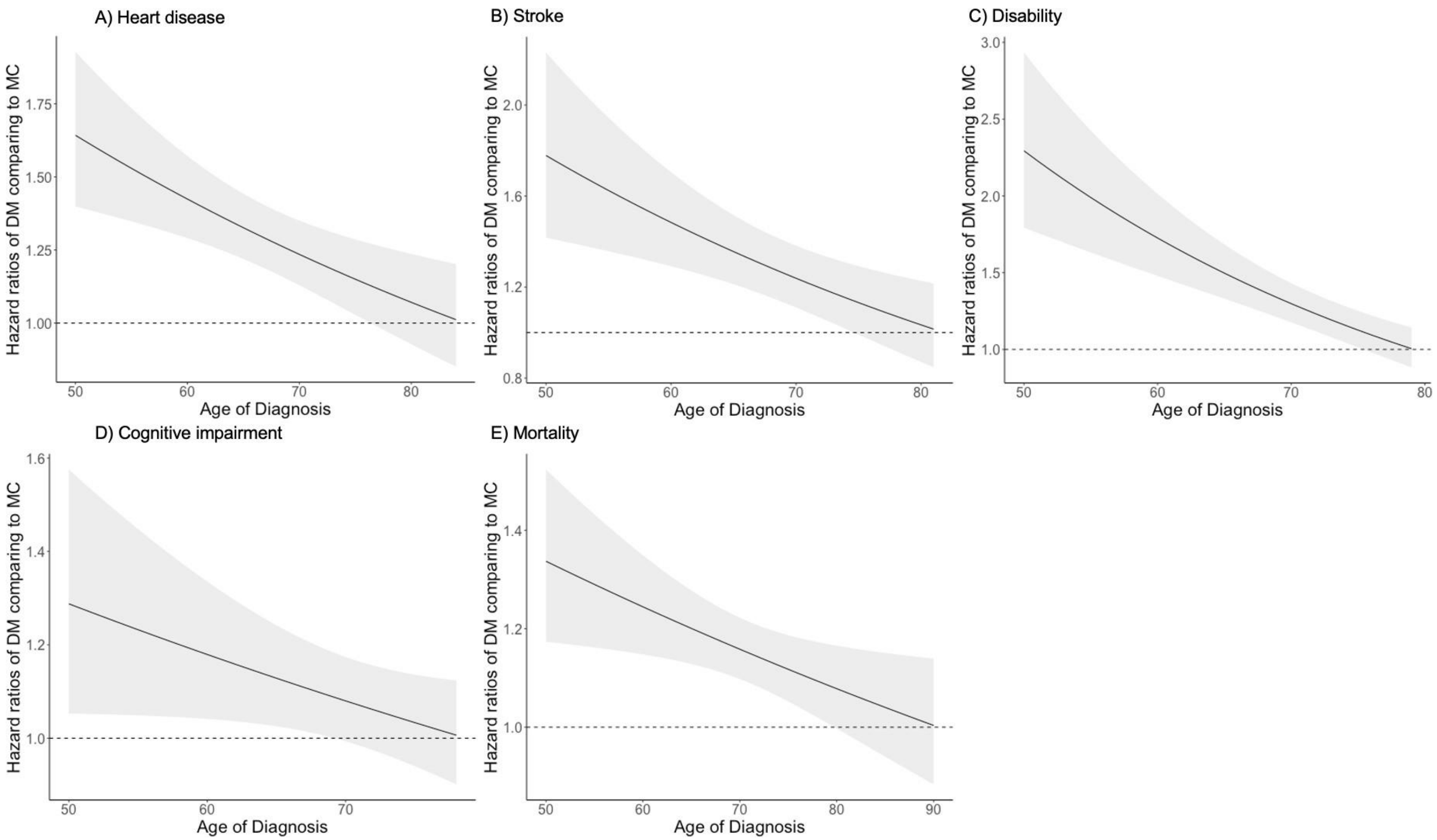

© 2022 Cigolle CT et al. *JAMA Network Open*.

The solid line denotes the hazard ratios (HRs) of diabetes on outcome and grey area denotes the corresponding 95% confident intervals. The dash line represents the null hypothesis (HR=1).

© 2022 Cigolle CT et al. *JAMA Network Open*.

eTable 1. Demographic Characteristics of Diabetes and Diabetes-Free Participants at the Year of Diabetes/Baseline

	Overall	Diabetes	Diabetes-free	P value[a]
N	36060	7739	28321	
Gender: Female, No. (%)	20388 (56.5)	4267 (55.1)	16121 (56.9)	0.01
Race/Ethnicity, No. (%)				<0.01
Hispanic	2330 (6.5)	726 (9.4)	1604 (5.7)	
Non-Hispanic Black	6480 (18.0)	1730 (22.4)	4750 (16.8)	
Non-Hispanic White	25310 (70.3)	4891 (63.3)	20419 (72.2)	
Other[b]	1893 (5.3)	383 (5.0)	1510 (5.3)	
Education, No. (%)				<0.01
<12	9525 (26.5)	2682 (34.8)	6843 (24.2)	
=12	11197 (31.1)	2425 (31.4)	8772 (31.1)	
>12	15233 (42.4)	2610 (33.8)	12623 (44.7)	
Cohort, No. (%)[c]				<0.01
AHEAD	6666 (18.5)	1394 (18.0)	5272 (18.6)	
CODA	3674 (10.2)	765 (9.9)	2909 (10.3)	
HRS	9450 (26.2)	2960 (38.2)	6490 (22.9)	
WB	3330 (9.2)	850 (11.0)	2480 (8.8)	
BB	12940 (35.9)	1770 (22.9)	11170 (39.4)	
At the year of diabetes onset/matching				
Age, mean (SD), y	62.89 (10.81)	67.42 (9.88)	61.66 (10.72)	<0.01
Marital status: Not Married or live alone, No. (%)	11453 (31.8)	2805 (36.2)	8648 (30.5)	<0.01
Wealth, $, No. (%)				<0.01
<40K	19266 (53.4)	4963 (64.1)	14303 (50.5)	
40~150K	13832 (38.4)	2420 (31.3)	11412 (40.3)	
150~300K	2271 (6.3)	280 (3.6)	1991 (7.0)	
>=300K	691 (1.9)	76 (1.0)	615 (2.2)	
BMI (median [IQR])[d]	26.60 [23.70, 30.60]	29.10 [25.70, 33.00]	26.30 [23.20, 29.70]	<0.01
Heart disease, No. (%)	6845 (19.0)	2584 (33.4)	4261 (15.0)	<0.01
Stroke, No. (%)	2207 (6.1)	901 (11.6)	1306 (4.6)	<0.01
Lung disease, No. (%)	3363 (9.3)	1012 (13.1)	2351 (8.3)	<0.01
Cancer, No. (%)	3638 (10.1)	1076 (13.9)	2562 (9.0)	<0.01
Arthritis, No. (%)	16608 (46.1)	4851 (62.7)	11757 (41.5)	<0.01
Cognition impairment, No. (%)				<0.01
Normal	28504 (79.0)	5579 (72.1)	22925 (80.9)	

© 2022 Cigolle CT et al. *JAMA Network Open*.

Minor impairment	5490 (15.2)	1454 (18.8)	4036 (14.3)	
Severe impairment	2066 (5.7)	706 (9.1)	1360 (4.8)	
During follow-up				
Death	13826 (38.3)	3555 (45.9)	10271 (36.3)	<0.01
Follow-up	12 [6, 22]	20 [8, 26]	8 [4, 20]	<0.01

a. P values are from ANOVA or rank sum test (for BMI) for comparing continuous covariates and the chi-square test for comparing categorical covariates

b. Other race/ethnicity includes American Indian, Alaskan Native, Asian, Native Hawaiian, and Pacific Islander, other race or unknown.

c. HRS included six birth cohorts: the Study of Assets and Health Dynamics Among the Oldest Old (AHEAD) cohort, born prior to 1924; the Children of the Depression (CODA) cohort, born 1924 to 1930; the original HRS cohort, born 1931 to 1941; the War Baby (WB) cohort, born 1942 to 1947; the Early Baby Boomer (EBB) cohort, born 1948 to 1953; and the Middle Baby Boomer (MBB) cohort, born 1954 to 1959.

d. BMI: body mass index, IQR: Interquartile range

© 2022 Cigolle CT et al. *JAMA Network Open*.

eTable 2. Demographic Characteristics of Each Diabetes Age-at-Diagnosis Group and Its Matched Control Group at the Year of Diabetes Diagnosis/Matching

A. Outcome: Heart Disease

	50-59			60-69			70+		
	Diabetes	MC	P value[a]	Diabetes	MC	P value[a]	Diabetes	MC	P value[a]
N before matching	1224	28321		1690	28321		1358	28321	
N after matching	1182	1182		1657	1657		1333	1333	
Gender: Female, No. (%)	731 (61.8)	703 (59.5)	0.26	874 (52.7)	874 (52.7)	1.00	779 (58.4)	744 (55.8)	0.18
Race/Ethnicity, No. (%)			0.42			0.53			0.59
Hispanic	186 (15.7)	188 (15.9)		182 (11.0)	158 (9.5)		105 (7.9)	88 (6.6)	
Non-Hispanic Black	323 (27.3)	291 (24.6)		399 (24.1)	398 (24.0)		258 (19.4)	267 (20.0)	
Non-Hispanic									
White	584 (49.4)	619 (52.4)		997 (60.2)	1014 (61.2)		924 (69.3)	936 (70.2)	
Other[b]	89 (7.5)	84 (7.1)		79 (4.8)	87 (5.3)		46 (3.5)	42 (3.2)	
Education, No. (%)			0.24			0.88			0.90
<12	347 (29.4)	328 (27.7)		548 (33.1)	537 (32.4)		518 (38.9)	507 (38.0)	
=12	361 (30.5)	399 (33.8)		530 (32.0)	542 (32.7)		430 (32.3)	438 (32.9)	
>12	474 (40.1)	455 (38.5)		579 (34.9)	578 (34.9)		385 (28.9)	388 (29.1)	
Cohort, No. (%)[c]			0.90			0.86			0.96
AHEAD	0 (0)	0 (0)		0 (0)	0 (0)		575 (43.1)	570 (42.8)	
CODA	0 (0)	0 (0)		103 (6.2)	99 (6.0)		279 (20.9)	272 (20.4)	
HRS	358 (30.3)	367 (31.0)		960 (57.9)	968 (58.4)		423 (31.7)	436 (32.7)	
WB	220 (18.6)	222 (18.8)		245 (14.8)	230 (13.9)		56 (4.2)	55 (4.1)	
BB	604 (51.1)	593 (50.2)		349 (21.1)	360 (21.7)		0 (0)	0 (0)	
At the year of diabetes onset/matching									
Age, mean (SD), y	55.55 (2.78)	55.54 (2.60)	0.95	64.16 (2.78)	64.10 (2.82)	0.51	76.79 (5.27)	76.74 (5.39)	0.83
Marital status: Not									
Married or live alone, No. (%)	288 (24.4)	260 (22.0)	0.19	491 (29.6)	462 (27.9)	0.28	613 (46.0)	604 (45.3)	0.76
Wealth, $, No. (%)			0.59			0.81			0.82

© 2022 Cigolle CT et al. *JAMA Network Open*.

<40K	602 (50.9)	582 (49.2)		946 (57.1)	932 (56.2)		998 (74.9)	1014 (76.1)	
40~150K	515 (43.6)	541 (45.8)		628 (37.9)	631 (38.1)		293 (22.0)	279 (20.9)	
150~300K	54 (4.6)	52 (4.4)		65 (3.9)	76 (4.6)		34 (2.6)	30 (2.3)	
>=300K	11 (0.9)	7 (0.6)		18 (1.1)	18 (1.1)		8 (0.6)	10 (0.8)	

© 2022 Cigolle CT et al. *JAMA Network Open*.

BMI (median [IQR])[d]	28.00 [26.60, 33.90]	27.40 [26.60, 33.30]	0.10	27.90 [26.60, 32.10]	27.10 [26.60, 32.30]	0.10	27.50 [24.70, 30.70]	27.20 [24.40, 30.80]	0.33
Stroke, No. (%)	40 (3.4)	28 (2.4)	0.18	102 (6.2)	87 (5.3)	0.29	145 (10.9)	135 (10.1)	0.57
Lung disease, No. (%)	86 (7.3)	80 (6.8)	0.69	145 (8.8)	149 (9.0)	0.86	105 (7.9)	94 (7.1)	0.46
Cancer, No. (%)	67 (5.7)	63 (5.3)	0.79	171 (10.3)	180 (10.9)	0.65	224 (16.8)	220 (16.5)	0.88
Arthritis, No. (%)	576 (48.7)	565 (47.8)	0.68	1029 (62.1)	1027 (62.0)	0.97	848 (63.6)	831 (62.3)	0.52
Cognition impairment, No. (%)			0.34			0.54			0.98
Normal	986 (83.4)	970 (82.1)		1299 (78.4)	1296 (78.2)		853 (64.0)	858 (64.4)	
Minor impairment	165 (14.0)	187 (15.8)		286 (17.3)	300 (18.1)		324 (24.3)	321 (24.1)	
Severe impairment	31 (2.6)	25 (2.1)		72 (4.3)	61 (3.7)		156 (11.7)	154 (11.6)	

a. P values are from ANOVA or rank sum test (for BMI) for comparing continuous covariates and the chi-square test for comparing categorical covariates

b. Other race/ethnicity includes American Indian, Alaskan Native, Asian, Native Hawaiian, and Pacific Islander, other race or unknown.

c. HRS included six birth cohorts: the Study of Assets and Health Dynamics Among the Oldest Old (AHEAD) cohort, born prior to 1924; the Children of the Depression (CODA) cohort, born 1924 to 1930; the original HRS cohort, born 1931 to 1941; the War Baby (WB) cohort, born 1942 to 1947; the Early Baby Boomer (EBB) cohort, born 1948 to 1953; and the Middle Baby Boomer (MBB) cohort, born 1954 to 1959.

d. BMI: body mass index, IQR: Interquartile range

© 2022 Cigolle CT et al. *JAMA Network Open*.

Supplementary Table 2B. Outcome: Stroke

		50-59			60-69			70+	
	Diabetes	MC	P value[a]	Diabetes	MC	P value[a]	Diabetes	MC	P value[a]
N before matching	1458	28321		2182	28321		1970	28321	
N after matching	1412	1412		2142	2142		1944	1944	
Gender: Female, No. (%)	868 (61.5)	848 (60.1)	0.46	1121 (52.3)	1049 (49.0)	0.03	1085 (55.8)	1120 (57.6)	0.27
Race/Ethnicity, No. (%)			0.59			0.89			0.99
Hispanic	204 (14.4)	209 (14.8)		205 (9.6)	197 (9.2)		124 (6.4)	123 (6.3)	
Non-Hispanic Black	373 (26.4)	363 (25.7)		495 (23.1)	506 (23.6)		322 (16.6)	314 (16.2)	
Non-Hispanic White	738 (52.3)	759 (53.8)		1347 (62.9)	1352 (63.1)		1440 (74.1)	1448 (74.5)	
Other[b]	97 (6.9)	81 (5.7)		95 (4.4)	87 (4.1)		58 (3.0)	59 (3.0)	
Education, No. (%)			0.10			0.73			0.88
<12	417 (29.5)	377 (26.7)		710 (33.1)	686 (32.0)		735 (37.8)	721 (37.1)	
=12	440 (31.2)	488 (34.6)		684 (31.9)	693 (32.4)		626 (32.2)	638 (32.8)	
>12	555 (39.3)	547 (38.7)		748 (34.9)	763 (35.6)		583 (30.0)	585 (30.1)	
Cohort, No. (%)[c]			0.75			0.80			0.96
AHEAD	0 (0)	0 (0)		0 (0)	0 (0)		850 (43.7)	854 (43.9)	
CODA	0 (0)	0 (0)		135 (6.3)	145 (6.8)		402 (20.7)	405 (20.8)	
HRS	443 (31.4)	460 (32.6)		1261 (58.9)	1261 (58.9)		619 (31.8)	618 (31.8)	
WB	261 (18.5)	263 (18.6)		327 (15.3)	308 (14.4)		73 (3.8)	67 (3.4)	
BB	708 (50.1)	689 (48.8)		419 (19.6)	428 (20.0)		0 (0)	0 (0)	
At the year of diabetes onset/matching									
Age, mean (SD), y	55.58 (2.75)	55.90 (2.45)	0.00	64.20 (2.79)	64.22 (2.74)	0.81	76.82 (5.24)	76.88 (5.26)	0.74
Marital status: Not Married or live alone, No. (%)	352 (24.9)	345 (24.4)	0.79	635 (29.6)	591 (27.6)	0.15	863 (44.4)	878 (45.2)	0.65
Wealth, $, No. (%)			0.51			0.59			0.99
<40K	726 (51.4)	714 (50.6)		1243 (58.0)	1263 (59.0)		1436 (73.9)	1428 (73.5)	
40~150K	609 (43.1)	630 (44.6)		792 (37.0)	786 (36.7)		449 (23.1)	455 (23.4)	
150~300K	64 (4.5)	61 (4.3)		87 (4.1)	71 (3.3)		46 (2.4)	48 (2.5)	
>=300K	13 (0.9)	7 (0.5)		20 (0.9)	22 (1.0)		13 (0.7)	13 (0.7)	

© 2022 Cigolle CT et al. *JAMA Network Open*.

BMI (median [IQR])[d]	27.80 [26.60, 33.73]	27.50 [26.60, 33.60]	0.42	27.90 [26.60, 32.30]	27.30 [26.60, 32.10]	0.02	27.70 [24.80, 31.10]	27.30 [24.40, 31.20]	0.07
Heart disease, No. (%)	270 (19.1)	274 (19.4)	0.89	587 (27.4)	574 (26.8)	0.68	756 (38.9)	748 (38.5)	0.82
Lung disease, No. (%)	148 (10.5)	153 (10.8)	0.81	256 (12.0)	254 (11.9)	0.96	222 (11.4)	233 (12.0)	0.62
Cancer, No. (%)	100 (7.1)	98 (6.9)	0.94	233 (10.9)	241 (11.3)	0.73	364 (18.7)	374 (19.2)	0.71
Arthritis, No. (%)	720 (51.0)	716 (50.7)	0.91	1385 (64.7)	1337 (62.4)	0.14	1307 (67.2)	1299 (66.8)	0.81
Cognition impairment, No. (%)			0.85			0.93			0.57
Normal	1174 (83.1)	1172 (83.0)		1695 (79.1)	1705 (79.6)		1275 (65.6)	1296 (66.7)	
Minor impairment	201 (14.2)	207 (14.7)		359 (16.8)	351 (16.4)		471 (24.2)	469 (24.1)	
Severe impairment	37 (2.6)	33 (2.3)		88 (4.1)	86 (4.0)		198 (10.2)	179 (9.2)	

a. P values are from ANOVA or rank sum test (for BMI) for comparing continuous covariates and the chi-square test for comparing categorical covariates

b. Other race/ethnicity includes American Indian, Alaskan Native, Asian, Native Hawaiian, and Pacific Islander, other race or unknown.

c. HRS included six birth cohorts: the Study of Assets and Health Dynamics Among the Oldest Old (AHEAD) cohort, born prior to 1924; the Children of the Depression (CODA) cohort, born 1924 to 1930; the original HRS cohort, born 1931 to 1941; the War Baby (WB) cohort, born 1942 to 1947; the Early Baby Boomer (EBB) cohort, born 1948 to 1953; and the Middle Baby Boomer (MBB) cohort, born 1954 to 1959.

d. BMI: body mass index, IQR: Interquartile range

© 2022 Cigolle CT et al. *JAMA Network Open*.

Supplementary Table 2C. Outcome: Disability

		50-59			60-69			70+	
	Diabetes	MC	P value[a]	Diabetes	MC	P value[a]	Diabetes	MC	P value[a]
N before matching	1509	28321		2318	28321		2189	28321	
N after matching	1462	1462		2257	2257		2133	2133	
Gender: Female, No.	892 (61.0)	858 (58.7)	0.21	1172	1128 (50.0)	0.20	1160 (54.4)	1166 (54.7)	0.88
(%)				(51.9)					
Race/Ethnicity, No. (%)			0.99			0.38			0.97
Hispanic	207 (14.2)	212 (14.5)		212 (9.4)	200 (8.9)		125 (5.9)	122 (5.7)	
Non-Hispanic Black	393 (26.9)	398 (27.2)		526 (23.3)	536 (23.7)		347 (16.3)	337 (15.8)	
Non-Hispanic White	760 (52.0)	751 (51.4)		1419	1442 (63.9)		1600 (75.0)	1614 (75.7)	
				(62.9)					
Other[b]	102 (7.0)	101 (6.9)		100 (4.4)	79 (3.5)		61 (2.9)	60 (2.8)	
Education, No. (%)			0.16			0.40			0.98
<12	438 (30.0)	410 (28.0)		744 (33.0)	703 (31.1)		784 (36.8)	789 (37.0)	
=12	458 (31.3)	506 (34.6)		722 (32.0)	752 (33.3)		708 (33.2)	709 (33.2)	
>12	566 (38.7)	546 (37.3)		791 (35.0)	802 (35.5)		641 (30.1)	635 (29.8)	
Cohort, No. (%)[c]			0.89			0.76			0.88
AHEAD	0 (0)	0 (0)		0 (0)	0 (0)		919 (43.1)	916 (42.9)	
CODA	0 (0)	0 (0)		141 (6.2)	153 (6.8)		452 (21.2)	472 (22.1)	
HRS	472 (32.3)	479 (32.8)		1328	1331 (59.0)		681 (31.9)	663 (31.1)	
				(58.8)					
WB	274 (18.7)	264 (18.1)		351 (15.6)	330 (14.6)		81 (3.8)	82 (3.8)	
BB	716 (49.0)	719 (49.2)		437 (19.4)	443 (19.6)		0 (0)	0 (0)	
At the year of diabetes o	set/matching								
Age, mean (SD), y	55.59	55.72 (2.51)	0.19	64.20	64.23 (2.75)	0.79	76.76 (5.14)	76.80 (5.14)	0.80
	(2.76)			(2.78)					
Marital status: Not Married or live alone, No. (%)	367 (25.1)	348 (23.8)	0.44	678 (30.0)	681 (30.2)	0.95	936 (43.9)	976 (45.8)	0.23
Wealth, $, No. (%)			0.66			0.85			0.83
<40K	766 (52.4)	750 (51.3)		1321	1320 (58.5)		1574 (73.8)	1589 (74.5)	
				(58.5)					
40~150K	618 (42.3)	645 (44.1)		826 (36.6)	814 (36.1)		492 (23.1)	474 (22.2)	

© 2022 Cigolle CT et al. *JAMA Network Open*.

150~300K	65 (4.4)	56 (3.8)		89 (3.9)	99 (4.4)		52 (2.4)	51 (2.4)	
>=300K	13 (0.9)	11 (0.8)		21 (0.9)	24 (1.1)		15 (0.7)	19 (0.9)	
BMI (median [IQR])[d]	27.70	27.00	0.07	27.90	27.30 [26.60,	0.04	27.50	27.20	0.01
	[26.60,	[26.60,		[26.60,	32.10]		[24.80,	[24.40,	
	33.70]	33.50]		32.30]			31.00]	30.70]	
Heart disease, No. (%)	302 (20.7)	294 (20.1)	0.75	647 (28.7)	608 (26.9)	0.21	871 (40.8)	900 (42.2)	0.38
Stroke, No. (%)	83 (5.7)	74 (5.1)	0.51	169 (7.5)	154 (6.8)	0.42	268 (12.6)	279 (13.1)	0.65
Lung disease, No. (%)	158 (10.8)	149 (10.2)	0.63	285 (12.6)	302 (13.4)	0.48	248 (11.6)	239 (11.2)	0.70
Cancer, No. (%)	98 (6.7)	96 (6.6)	0.94	245 (10.9)	239 (10.6)	0.81	409 (19.2)	401 (18.8)	0.79
Arthritis, No. (%)	748 (51.2)	711 (48.6)	0.18	1462	1455 (64.5)	0.85	1429 (67.0)	1451 (68.0)	0.49
				(64.8)					
Cognition impairment,			0.95			0.63			0.97
No. (%)									
Normal	1221	1226 (83.9)		1786	1803 (79.9)		1418 (66.5)	1412 (66.2)	
	(83.5)			(79.1)					
Minor impairment	211 (14.4)	208 (14.2)		389 (17.2)	383 (17.0)		538 (25.2)	545 (25.6)	
Severe impairment	30 (2.1)	28 (1.9)		82 (3.6)	71 (3.1)		177 (8.3)	176 (8.3)	

a. P values are from ANOVA or rank sum test (for BMI) for comparing continuous covariates and the chi-square test for comparing categorical covariates

b. Other race/ethnicity includes American Indian, Alaskan Native, Asian, Native Hawaiian, and Pacific Islander, other race or unknown.

c. HRS included six birth cohorts: the Study of Assets and Health Dynamics Among the Oldest Old (AHEAD) cohort, born prior to 1924; the Children of the Depression (CODA) cohort, born 1924 to 1930; the original HRS cohort, born 1931 to 1941; the War Baby (WB) cohort, born 1942 to 1947; the Early Baby Boomer (EBB) cohort, born 1948 to 1953; and the Middle Baby Boomer (MBB) cohort, born 1954 to 1959.

d. BMI: body mass index, IQR: Interquartile range

© 2022 Cigolle CT et al. *JAMA Network Open*.

Supplementary Table 2D. Outcome: Severe Cognitive Impairment

	50-59			60-69			70+		
	Diabetes	MC	P value[a]	Diabetes	MC	P value[a]	Diabetes	MC	P value[a]
N before matching	1495	28321		2207	28321		1937	28321	
N after matching	1450	1450		2165	2165		1915	1915	
Gender: Female, No. (%)	885 (61.0)	862 (59.4)	0.40	1123 (51.9)	1098 (50.7)	0.47	1039 (54.3)	1049 (54.8)	0.77
Race/Ethnicity, No. (%)			0.51			0.60			0.57
Hispanic	200 (13.8)	205 (14.1)		199 (9.2)	185 (8.5)		105 (5.5)	110 (5.7)	
Non-Hispanic Black	390 (26.9)	365 (25.2)		471 (21.8)	497 (23.0)		266 (13.9)	242 (12.6)	
Non-Hispanic White	761 (52.5)	793 (54.7)		1399 (64.6)	1398 (64.6)		1493 (78.0)	1519 (79.3)	
Other[b]	99 (6.8)	87 (6.0)		96 (4.4)	85 (3.9)		51 (2.7)	44 (2.3)	
Education, No. (%)			0.77			0.67			0.77
<12	422 (29.1)	433 (29.9)		662 (30.6)	635 (29.3)		614 (32.1)	627 (32.7)	
=12	455 (31.4)	463 (31.9)		707 (32.7)	720 (33.3)		672 (35.1)	651 (34.0)	
>12	573 (39.5)	554 (38.2)		796 (36.8)	810 (37.4)		629 (32.8)	637 (33.3)	
Cohort, No. (%)[c]			0.20			0.65			0.99
AHEAD	0 (0)	0 (0)		0 (0)	0 (0)		817 (42.7)	806 (42.1)	
CODA	0 (0)	0 (0)		138 (6.4)	154 (7.1)		402 (21.0)	408 (21.3)	
HRS	470 (32.4)	498 (34.3)		1262 (58.3)	1239 (57.2)		624 (32.6)	630 (32.9)	
WB	275 (19.0)	240 (16.6)		337 (15.6)	326 (15.1)		72 (3.8)	71 (3.7)	
BB	705 (48.6)	712 (49.1)		428 (19.8)	446 (20.6)		0 (0)	0 (0)	
At the year of diabetes onset/matching									
Age, mean (SD), y	55.58 (2.76)	55.67 (2.59)	0.33	64.15 (2.77)	64.13 (2.76)	0.77	76.59 (5.07)	76.41 (4.80)	0.26
Marital status: Not Married or live alone, No. (%)	361 (24.9)	361 (24.9)	1.00	629 (29.1)	630 (29.1)	1.00	821 (42.9)	824 (43.0)	0.95
Wealth, $, No. (%)			0.91			0.85			0.82
<40K	751 (51.8)	765 (52.8)		1243 (57.4)	1250 (57.7)		1382 (72.2)	1407 (73.5)	
40~150K	621 (42.8)	612 (42.2)		812 (37.5)	796 (36.8)		467 (24.4)	448 (23.4)	
150~300K	65 (4.5)	59 (4.1)		89 (4.1)	93 (4.3)		51 (2.7)	47 (2.5)	
>=300K	13 (0.9)	14 (1.0)		21 (1.0)	26 (1.2)		15 (0.8)	13 (0.7)	

© 2022 Cigolle CT et al. *JAMA Network Open*.

BMI (median [IQR])[d]	27.40	27.20	0.21	27.90	27.40	0.14	27.60	27.40	0.37
	[26.60,	[26.60,		[26.60,	[26.60,		[24.90,	[24.70,	
	33.70]	33.50]		32.30]	32.30]		31.00]	30.90]	
Heart disease, No. (%)	307 (21.2)	303 (20.9)	0.89	621 (28.7)	585 (27.0)	0.24	791 (41.3)	785 (41.0)	0.87
Stroke, No. (%)	88 (6.1)	84 (5.8)	0.81	160 (7.4)	152 (7.0)	0.68	231 (12.1)	223 (11.6)	0.73
Lung disease, No. (%)	157 (10.8)	162 (11.2)	0.81	279 (12.9)	277 (12.8)	0.96	233 (12.2)	232 (12.1)	1.00
Cancer, No. (%)	98 (6.8)	96 (6.6)	0.94	244 (11.3)	231 (10.7)	0.56	375 (19.6)	380 (19.8)	0.87
Arthritis, No. (%)	742 (51.2)	729 (50.3)	0.66	1407 (65.0)	1370 (63.3)	0.25	1286 (67.2)	1305 (68.1)	0.53

a. P values are from ANOVA or rank sum test (for BMI) for comparing continuous covariates and the chi-square test for comparing categorical covariates

b. Other race/ethnicity includes American Indian, Alaskan Native, Asian, Native Hawaiian, and Pacific Islander, other race or unknown.

c. HRS included six birth cohorts: the Study of Assets and Health Dynamics Among the Oldest Old (AHEAD) cohort, born prior to 1924; the Children of the Depression (CODA) cohort, born 1924 to 1930; the original HRS cohort, born 1931 to 1941; the War Baby (WB) cohort, born 1942 to 1947; the Early Baby Boomer (EBB) cohort, born 1948 to 1953; and the Middle Baby Boomer (MBB) cohort, born 1954 to 1959.

d. BMI: body mass index, IQR: Interquartile range

© 2022 Cigolle CT et al. *JAMA Network Open*.

Supplementary Table 2E. Outcome: All-cause mortality

	50-59			60-69			70+		
	Diabetes	MC	P value[a]	Diabetes	MC	P value[a]	Diabetes	MC	P value[a]
N before matching	1552	28321		2388	28321		2310	28321	
N after matching	1504	1504		2343	2343		2278	2278	
Gender: Female, No. (%)	923 (61.4)	896 (59.6)	0.33	1227 (52.4)	1177 (50.2)	0.15	1261 (55.4)	1250 (54.9)	0.77
Race/Ethnicity, No. (%)			0.86			0.25			0.59
Hispanic	214 (14.2)	223 (14.8)		224 (9.6)	195 (8.3)		138 (6.1)	155 (6.8)	
Non-Hispanic Black	411 (27.3)	406 (27.0)		553 (23.6)	556 (23.7)		391 (17.2)	366 (16.1)	
Non-Hispanic White	774 (51.5)	780 (51.9)		1461 (62.4)	1504 (64.2)		1683 (73.9)	1694 (74.4)	
Other[b]	105 (7.0)	95 (6.3)		105 (4.5)	88 (3.8)		66 (2.9)	63 (2.8)	
Education, No. (%)			0.62			0.14			0.73
<12	458 (30.5)	437 (29.1)		791 (33.8)	755 (32.2)		873 (38.3)	878 (38.5)	
=12	465 (30.9)	486 (32.3)		743 (31.7)	807 (34.4)		739 (32.4)	716 (31.4)	
>12	581 (38.6)	581 (38.6)		809 (34.5)	781 (33.3)		666 (29.2)	684 (30.0)	
Cohort, No. (%)[c]			0.55			0.66			0.72
AHEAD	0 (0)	0 (0)		0 (0)	0 (0)		1009 (44.3)	1007 (44.2)	
CODA	0 (0)	0 (0)		150 (6.4)	162 (6.9)		475 (20.9)	494 (21.7)	
HRS	483 (32.1)	504 (33.5)		1381 (58.9)	1374 (58.6)		712 (31.3)	707 (31.0)	
WB	282 (18.8)	262 (17.4)		357 (15.2)	334 (14.3)		82 (3.6)	70 (3.1)	
BB	739 (49.1)	738 (49.1)		455 (19.4)	473 (20.2)		0 (0)	0 (0)	
At the year of diabetes onset/matching									
Age, mean (SD), y	55.59 (2.75)	55.70 (2.52)	0.25	64.21 (2.78)	64.21 (2.83)	0.94	77.09 (5.38)	77.20 (5.28)	0.47
Marital status: Not Married or live alone, No. (%)	381 (25.3)	381 (25.3)	1.00	713 (30.4)	672 (28.7)	0.20	1034 (45.4)	1059 (46.5)	0.48
Wealth, $, No. (%)			0.83			0.52			0.91
<40K	801 (53.3)	784 (52.1)		1402 (59.8)	1369 (58.4)		1710 (75.1)	1731 (76.0)	

© 2022 Cigolle CT et al. *JAMA Network Open*.

40~150K	625 (41.6)	645 (42.9)		831 (35.5)	853 (36.4)		500 (21.9)	483 (21.2)	

© 2022 Cigolle CT et al. *JAMA Network Open*.

150~300K	65 (4.3)	65 (4.3)		89 (3.8)	104 (4.4)		53 (2.3)	50 (2.2)	
>=300K	13 (0.9)	10 (0.7)		21 (0.9)	17 (0.7)		15 (0.7)	14 (0.6)	
BMI (median [IQR])[d]	27.50 [26.60,	27.40	0.35	27.90	27.30	0.01	27.50	27.30	0.03
	33.70]	[26.60,		[26.60,	[26.60,		[24.80,	[24.40,	
		33.70]		32.30]	32.10]		31.00]	30.70]	
Heart disease, No. (%)	322 (21.4)	323 (21.5)	1.00	686 (29.3)	650 (27.7)	0.26	945 (41.5)	969 (42.5)	0.49
Stroke, No. (%)	92 (6.1)	76 (5.1)	0.23	201 (8.6)	165 (7.0)	0.06	334 (14.7)	332 (14.6)	0.97
Lung disease, No. (%)	168 (11.2)	171 (11.4)	0.91	304 (13.0)	302 (12.9)	0.97	267 (11.7)	246 (10.8)	0.35
Cancer, No. (%)	103 (6.8)	83 (5.5)	0.15	254 (10.8)	243 (10.4)	0.64	429 (18.8)	411 (18.0)	0.52
Arthritis, No. (%)	782 (52.0)	771 (51.3)	0.72	1535 (65.5)	1517 (64.7)	0.60	1546	1561 (68.5)	0.66
							(67.9)		
Cognition impairment, No. (%)			0.43			0.61			0.83
Normal	1235 (82.1)	1259 (83.7)		1818 (77.6)	1807 (77.1)		1438	1451 (63.7)	
							(63.1)		
Minor impairment	228 (15.2)	203 (13.5)		406 (17.3)	427 (18.2)		556 (24.4)	556 (24.4)	
Severe impairment	41 (2.7)	42 (2.8)		119 (5.1)	109 (4.7)		284 (12.5)	271 (11.9)	

a. P values are from ANOVA or rank sum test (for BMI) for comparing continuous covariates and the chi-square test for comparing categorical covariates
b. Other race/ethnicity includes American Indian, Alaskan Native, Asian, Native Hawaiian, and Pacific Islander, other race or unknown.
c. HRS included six birth cohorts: the Study of Assets and Health Dynamics Among the Oldest Old (AHEAD) cohort, born prior to 1924; the Children of the Depression (CODA) cohort, born 1924 to 1930; the original HRS cohort, born 1931 to 1941; the War Baby (WB) cohort, born 1942 to 1947; the Early Baby Boomer (EBB) cohort, born 1948 to 1953; and the Middle Baby Boomer (MBB) cohort, born 1954 to 1959.
d. BMI: body mass index, IQR: Interquartile range

© 2022 Cigolle CT et al. *JAMA Network Open*.
